# Deciphering essential cistromes using genome-wide CRISPR screens

**DOI:** 10.1073/pnas.1908155116

**Published:** 2019-11-14

**Authors:** Teng Fei, Wei Li, Jingyu Peng, Tengfei Xiao, Chen-Hao Chen, Alexander Wu, Jialiang Huang, Chongzhi Zang, X. Shirley Liu, Myles Brown

**Affiliations:** ^a^College of Life and Health Sciences, Northeastern University, 110819 Shenyang, People’s Republic of China;; ^b^Key Laboratory of Data Analytics and Optimization for Smart Industry, Northeastern University, Ministry of Education, 110819 Shenyang, People’s Republic of China;; ^c^Division of Molecular and Cellular Oncology, Dana-Farber Cancer Institute, Boston, MA 02215;; ^d^Department of Medical Oncology, Dana-Farber Cancer Institute, Boston, MA 02215;; ^e^Center for Functional Cancer Epigenetics, Dana-Farber Cancer Institute, Boston, MA 02215;; ^f^Department of Medicine, Brigham and Women’s Hospital and Harvard Medical School, Boston, MA 02215;; ^g^Department of Data Sciences, Dana-Farber Cancer Institute and Harvard T.H. Chan School of Public Health, Boston, MA 02215;; ^h^Center for Genetic Medicine Research, Children’s National Hospital, Washington, DC 20010;; ^i^Department of Genomics and Precision Medicine, The George Washington School of Medicine and Health Sciences, Washington, DC 20010;; ^j^Program in Computational Biology and Quantitative Genetics, Harvard School of Public Health, Boston, MA 02215;; ^k^Center for Public Health Genomics, University of Virginia, Charlottesville, VA 22908

**Keywords:** CRISPR screen, cistrome, CTCF, FOXA1, enhancer

## Abstract

Although millions of transcription factor binding sites, or cistromes, have been identified across the human genome, defining which of these sites is functional in a given condition remains challenging. Using CRISPR/Cas9 knockout screens and gene essentiality or fitness as the readout, we systematically investigated the essentiality of over 10,000 FOXA1 and CTCF binding sites in breast and prostate cancer cells. We found that essential FOXA1 binding sites act as enhancers to orchestrate the expression of nearby essential genes through the binding of lineage-specific transcription factors. In contrast, CRISPR screens of the CTCF cistrome revealed 2 classes of essential binding sites. The first class of essential CTCF binding sites act like FOXA1 sites as enhancers to regulate the expression of nearby essential genes, while a second class of essential CTCF binding sites was identified at topologically associated domain (TAD) boundaries and display distinct characteristics. Using regression methods trained on our screening data and public epigenetic profiles, we developed a model to predict essential *cis*-elements with high accuracy. The model for FOXA1 essentiality correctly predicts noncoding variants associated with cancer risk and progression. Taken together, CRISPR screens of *cis*-regulatory elements can define the essential cistrome of a given factor and can inform the development of predictive models of cistrome function.

## Significance

Systematically dissecting the function of a large set of *cis*-regulatory elements or transcription factor binding sites (cistromes) has been technically challenging. Using genome-wide CRISPR screens, we profiled over 10,000 FOXA1 and CTCF binding sites for their roles in regulating the fitness of breast and prostate cancer cells, and accordingly developed a model to predict essentiality for *cis*-elements. These efforts not only reveal how the key transcription factors and their cistromes regulate cell essentiality in hormone-dependent cancers but also highlight an efficient approach to investigate the functions of noncoding regions of the genome.

Gene expression in mammalian systems is exquisitely regulated by the combinatorial action of a set of transacting factors and their “cistromes” or genome-wide *cis*-acting genomic targets. Cistromes include many important regulatory elements in the genome, such as promoters, enhancers, silencers, or insulators. Enhancers are thought to be the most abundant, and enhancer selectivity and activity may determine the action of a transcription factor in a cell type-dependent manner. Enhancer abnormalities contribute to a variety of human diseases, including cancer (1, 2). Millions of putative enhancers have been found in the human genome through high-throughput profiling histone modification, transcription factor binding sites, and chromatin accessibility (3–5). However, given the surge of cistrome data generation and enhancer characterization, it remains challenging to distinguish functional binding sites or enhancers from passive binding events or inactive enhancers. In addition, the extent of functional redundancy of enhancers is unknown. Recently, high-throughput CRISPR/Cas9 genetic screening utilizing single-guide RNAs (sgRNAs) or paired-guide RNAs (pgRNAs) have been applied to characterize noncoding genomic region functions (6–8). These studies either interrogate a genomic region close to the gene of interest to identify enhancers that regulate the target gene (6, 9–11), or perturb hundreds of enhancers and test their knockout effects on cell growth (12, 13). However, to systematically evaluate cistrome functions, these current approaches are limited since 1) most of the studies focus on the regulation of only 1 gene; 2) the number of noncoding genomic regions is limited (only up to a few hundred); and 3) a systematic evaluation of the screening results and associated features is lacking.

Here we interrogated the functions of over 10,000 *cis*-acting elements that are bound by CTCF or FOXA1 using CRISPR sgRNA screens and delineated binding site essentiality or fitness. FOXA1 (forkhead box protein A1) is a pioneer factor that is thought to open chromatin and promote gene transcription through binding of other factors (14, 15). CTCF (CCCTC-binding factor) is a highly conserved factor with diverse functions in mammalian cells, including transcriptional activation or repression (16, 17), imprinting (18), insulation (19), and chromatin organization (20). Both factors as well as their binding sites are frequently mutated in different cancer types (21–23). We validated top hits through a focused pgRNA screen and individual knockout, identified features associated with the essential sites within the cistromes, and built machine-learning methods to predict the essentiality of *cis-*acting elements.

## Results

### Genome-Wide CRISPR Screen Targeting the FOXA1 Cistrome.

**Fig. 1.**
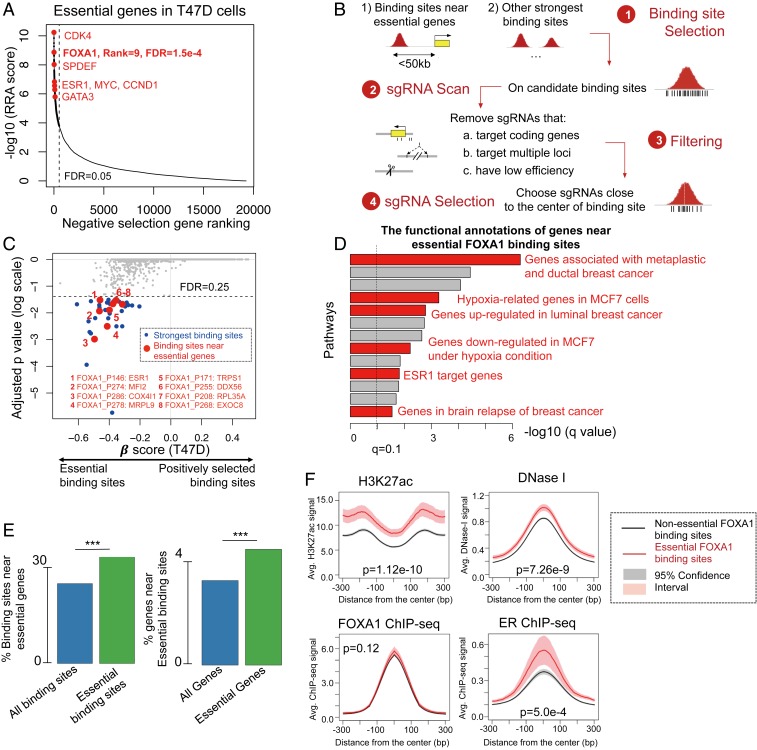
Genome-wide CRISPR screens for FOXA1 binding sites in T47D cells, an ER-positive breast cancer cell line. (*A*) Top essential genes in T47D cells, identified by genome-wide CRISPR gene screens. A smaller RRA score (identified by the MAGeCK algorithm) indicates a stronger negative selection of the corresponding gene. (*B*) The design of the FOXA1 screening library. FOXA1 binding sites are preselected as indicated, followed by an sgRNA scanning to identify all possible guides within the binding sites. sgRNAs that have low predicted specificity or cleavage efficacy are then filtered. For the remaining sgRNAs, up to 20 guides that are close to the binding site summit are then selected. (*C*) An overview of the functions of FOXA1 binding sites in T47D cells, including strong intergenic binding sites (blue dots) and essential binding sites near essential genes (red dots). For each binding site, the β-score, a measurement of gene selection in the screen, is calculated using the MAGeCK-VISPR algorithm that we previously developed. A positive (or negative) β-score indicates the gene/binding site is positively (or negatively) selected, respectively. (*D*) The functional analysis of genes near essential FOXA1 binding sites using the GREAT prediction tool (46). Terms related to breast cancer are highlighted in red. (*E*) The percentage of all (and essential) FOXA1 binding sites that are within 100 kb of essential genes in T47D cells, and the percentage of all (and essential) genes near essential FOXA1 binding sites. Essential genes are genes with the 10% lowest β-scores from genome-wide CRISPR gene screens in T47D cells. ***P < 0.001. (*F*) The epigenetic features of essential binding sites vs. nonessential binding sites.

To systematically investigate sites in the FOXA1 cistrome whose loss affects cell viability, we designed genome-wide CRISPR/Cas9 knockout screening libraries targeting the binding sites of FOXA1. We first performed genome-wide CRISPR gene screens in estrogen receptor (ER) positive breast cancer T47D cells with our optimized sgRNA library targeting ∼18,000 genes in the human genome (*SI Appendix*, *Methods*). The quality of the screens is high based on multiple quality control (QC) measurements (*SI Appendix*, Fig. S1 *A*–*C*), and the results confirmed FOXA1 as an essential gene in T47D cells (Fig. 1*A* and *SI Appendix*, Table S1). Over 500 genes are identified as essential with high statistical significance (false discovery rate [FDR] < 0.05), including genes in the ER transcription factor network such as ESR1, FOXA1, TRPS1, GATA3, and SPDEF and known essential target genes and downstream signaling molecules such as MYC, CCND1, and CDK4 (Fig. 1*A* and *SI Appendix*, Table S1). In order to design an sgRNA library to interrogate the FOXA1 cistrome, we focused on all 1,122 binding sites within 50 kb of essential genes in T47D and chose 5,000 FOXA1 binding sites that have the strongest chromatin immunoprecipitation-sequencing (ChIP-seq) signals (Fig. 1*B*, Table 1, and *SI Appendix*, Tables S1 and S2), identified from either our gene screen or a public CRISPR screen of T47D cells that employed the GeCKO v2 library (24) (*SI Appendix*, Fig. S1*B*). All of the selected binding sites are located within the intronic or intergenic regions of the genome (*SI Appendix*, Fig. S1*D*). For candidate FOXA1 binding sites, we next scanned for all possible sgRNAs located within the FOXA1 ChIP-seq peak, removed those with low predicted CRISPR cutting efficiency and specificity, and chose up to 20 sgRNAs that were closest to the ChIP-seq peak summit. Also included in the FOXA1 cistrome library are sgRNAs targeting the exons of known essential genes as positive controls and the nonessential AAVS1 “safe harbor” locus as a negative control. We performed CRISPR/Cas9 screening in T47D cells under full medium condition to evaluate the essentiality of the selected FOXA1 binding sites (*SI Appendix*, Fig. S1*E*). The sequences encoding the sgRNA were PCR-amplified from the transduced cells at day 0 and after 4 wk of culture. The abundance of sgRNAs was then quantified by high-throughput sequencing, and the data analysis was performed using MAGeCK-VISPR, a statistical algorithm that we previously developed (25, 26).

**Table 1.** A summary of the CTCF and FOXA1 cistrome-targeting libraries

	FOXA1 library	CTCF library	Total
Binding sites	6,110	5,564	11,674
sgRNAs (12 to 20 sgRNAs per binding site)	96,962	97,002	193,964
Essential genes	146	146	
Gene-targeting sgRNAs (5 sgRNAs per gene)	730	730	
AAVS1-targeting sgRNAs	267	267	

We compared the degree of essentiality for genes or binding sites using the “β-score” generated from MAGeCK-VISPR, which is a measurement of gene selection similar to the term “log fold change” in differential expression analysis (*SI Appendix*, Table S3). A positive (or negative) β-score indicates the corresponding gene/binding site is under positive (or negative) selection in the CRISPR screen, respectively. Overall, essential genes that serve as positive controls were strongly negatively selected as expected (*SI Appendix*, Fig. S1*F*). Cell type-specific copy-number variation (CNV) is a well-known confounding factor in CRISPR knockout screens. We employed the CNV correction procedure in MAGeCK to reduce the effects of CNV, by adjusting the β-scores according to the CNV status in the corresponding loci (*SI Appendix*, *Methods*). This approach reduces the effects of CNV status and allows the accurate evaluation of the functions of binding sites within amplified regions (*SI Appendix*, Fig. S1*G*).

Overall, 37 FOXA1 binding sites in T47D cells are essential with statistical significance (FDR < 0.25; Fig. 1*C*), including 29 strong FOXA1 binding sites and 8 binding sites near essential genes [gene FDR < 0.05 in either our screen or a public T47D cell screen (24); *SI Appendix*, Fig. S1*B*]. Essential genes associated with essential binding sites include estrogen receptor 1 (ESR1), the master transcription factor for ER+ breast cancer cells, and TRPS1, another transcription factor that is known to be associated with ER+ breast cancer progression (27). In addition, binding sites near genes that are widely essential for cell growth were also found in the screen, including for example MRPL9, a mitochondrial large ribosomal subunit protein, and COX4I1, a cytochrome *C* oxidase subunit.

As one of the top essential FOXA1 binding sites (FOXA1_P146) in T47D cells is located within the intron of the *ESR1* gene itself (*SI Appendix*, Fig. S2*A*), we chose it for validation. Using 2 independent sgRNAs targeting this binding site, we confirmed that they induced a high percentage of indels at the binding site (*SI Appendix*, Fig. S3 *A* and *B*), and the CRISPR/Cas9–mediated mutagenesis of the FOXA1_P146 binding site decreased cell proliferation in a competitive growth assay (*SI Appendix*, Fig. S3*C*). Disruption of this binding site by the individual sgRNA-mediated CRISPR/Cas9 targeting compromises binding of FOXA1 to the site (*SI Appendix*, Fig. S3*D*). We further examined whether FOXA1_P146 could regulate *ESR1* expression, considering the localization of this binding site and the functional importance of *ESR1* in T47D cells. Individual targeting of FOXA1_P146 reduces *ESR1* expression, which is similar to the effect of FOXA1 loss of function by either sgRNA-mediated CRISPR/Cas9 knockout or RNA interference (*SI Appendix*, Fig. S3 *E* and *F*), suggesting that FOXA1_P146 serves as a FOXA1-dependent enhancer of ESR1 expression.

We next studied the link between essential binding sites and nearby genes (Fig. 1 *D* and *E*), as well as epigenetic features associated with essential binding sites (Fig. 1*F*). In general, essential binding sites are more likely to be close to essential genes (compared with all binding sites in the library) and essential genes are more likely to be located near essential binding sites compared with all genes (Fig. 1*E*). Those genes near essential FOXA1 binding sites are enriched in breast cancer-related functions and pathways, including metaplastic/ductal carcinoma of the breast, luminal breast cancer, hypoxia response in MCF7 cells, and targets of ESR1 (Fig. 1*D*). Compared with all binding sites in the library, essential FOXA1 binding sites tend to have a higher level of H3K27ac signal (consistent with an active enhancer) and DNase I hypersensitivity signals (indicating open chromatin; Fig. 1*F*). However, essential binding sites have a similar level of FOXA1 binding strength compared with nonessential binding sites, indicating that the intensity of FOXA1 binding is not necessarily associated with essentiality.

**Fig. 2.**
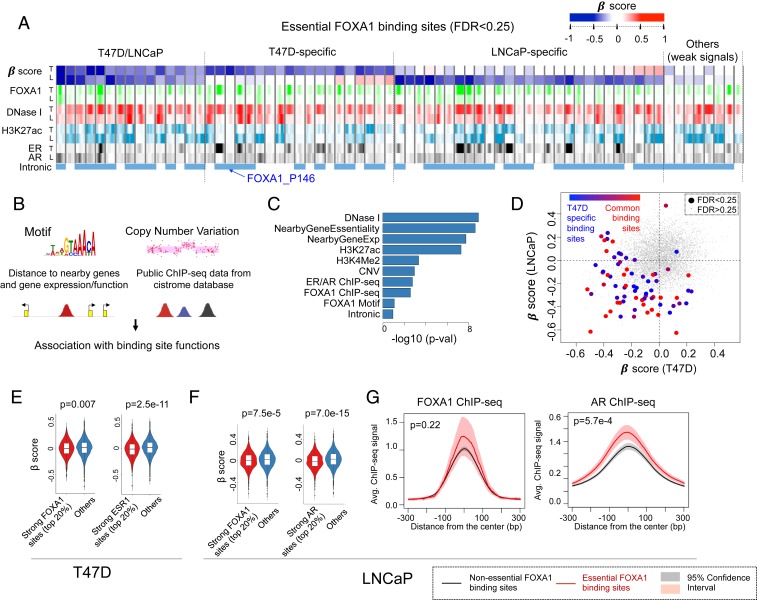
Features of FOXA1 binding sites in T47D and LNCaP cells. (*A*) The chromatin features of selected binding sites with statistical significance coming out of the cistrome screens. (*B*) Possible features that are tested for the association with binding site functions in the screens. (*C*) The rankings of all features associated with the functions of FOXA1 binding sites. For each feature, we compare its signal distribution between the top 5% of essential sites vs. other sites. The average of *P* values (calculated using the Mann–Whitney *U* test) across 2 cell lines is used to measure the relevance of each feature. (*D*) The β-scores of all sites in T47D and LNCaP cells. Sites are colored by their appearances in both cell lines: Sites that only appear in T47D or appear in both cells are colored blue and red, respectively. (*E* and *F*) The β-score distribution of the strongest FOXA1 or ESR1 sites vs. others in T47D and LNCaP cells. (*G*) The binding signals of the top essential FOXA1 binding sites vs. nonessential sites in LNCaP cells.

To gain insights into essential binding sites in a different cell lineage, we performed the same screening experiment in the LNCaP prostate cancer cell line, where FOXA1 is also known to be essential (*SI Appendix*, Fig. S4 *A*–*C*). The quality of the screen was confirmed by evaluating the positive control genes that are strongly negatively selected (*SI Appendix*, Fig. S4*D*). Together, both T47D and LNCaP screens identified 72 essential binding sites in at least 1 cell line (FDR < 0.25; Fig. 2*A*). Interestingly, many of the essential binding sites are located within the introns of a protein-coding gene (last row, Fig. 2*A*). The β-scores of intronic binding sites and their associated genes are positively correlated (*SI Appendix*, Fig. S4*E*). However, it was unclear whether these intronic binding sites would affect gene functions via enhancer regulation or other mechanisms (e.g., RNA stability or splicing). To address this question, we examined the H3K27ac levels (an enhancer mark) of intronic binding sites, as well as their distances to the transcription start site (TSS) of genes (*SI Appendix*, Fig. S4 *F* and *G*). Overall, these “intronic” essential binding sites have similarly higher H3K27ac levels as intergenic essential binding sites compared with all of the tested binding sites, and binding sites closer to the gene TSS are more likely to be essential (*SI Appendix*, Fig. S4 *F* and *G*). When only focusing on intronic sites, the essential intronic sites also have stronger signals for enhancer-associated chromatin features including H3K4me2, H3K27ac, DNase I sensitivity, and ER binding over the nonessential intronic sites (*SI Appendix*, Fig. S4 *H* and *I*). These results support that overall the essentiality of intronic sites is likely the result of their function as enhancers rather than effects on the primary transcript, though this possibility for any individual site cannot be ruled out without further validation.

We set out to systematically evaluate features associated with FOXA1 binding site essentiality. We collected features that are potentially associated with binding site functions (Fig. 2*B*), including the locations of the FOXA1 motif, copy-number status, distance of binding sites to nearby genes, expression of nearby genes (through RNA-seq), essentiality of nearby genes (through CRISPR gene screens), as well as high-quality transcription factor and histone mark ChIP-seq data corresponding to both cell lines from our cistrome database (28, 29). We ranked features based on their associations with functional or nonfunctional binding sites identified in both T47D and LNCaP cell screens, using the Wilcoxon rank-sum test (Fig. 2*C*). Overall, DNase I signal (representing open chromatin), nearby gene essentiality, and nearby gene expression have the strongest associations with binding site essentiality. Top essential binding sites are significantly closer to essential genes (Fig. 1*E* and *SI Appendix*, Fig. S5*A*) and highly expressed genes (*SI Appendix*, Fig. S5*B*), suggesting that these essential binding sites may function through regulating nearby essential and often highly expressed genes. As expected, H3K27ac and DNase I signals also separate essential and nonessential binding sites (Fig. 1*F* and *SI Appendix*, Fig. S5*C*), indicating that functional binding sites tend to be active enhancers.

Consistent with our observation (Fig. 1*F*) that FOXA1 binding strength is weakly associated with essentiality (Fig. 2*C*), binding sites identified as being T47D-specific by virtue of strong FOXA1 binding in T47D cells may also be essential in LNCaP cells (Fig. 2*D*). This may be because FOXA1 acts as a pioneer factor to open chromatin and allow other transcription factors to bind the *cis*-regulatory region to regulate gene expression. Thus, it may not be FOXA1 itself but other key cooperative transcription factors that determine the function of the binding sites. Indeed, ER or androgen receptor (AR) binding strength, rather than FOXA1 binding, is more predictive of functional binding sites in T47D (Figs. 1*F* and 2*E*) and LNCaP cells (Fig. 2 *F* and *G*), respectively. Conversely, essential binding sites are more likely to have stronger ER or AR binding. We confirmed our finding by performing screens in hormone-depleted media and hormone-depleted media supplemented with 17β-estradiol (E2) in T47D cells (*SI Appendix*, Fig. S6*A*). Compared with other binding sites, binding sites with the strongest ER binding (after E2 induction) appear to be strongly negatively selected, whereas binding sites with the strongest FOXA1 binding (after E2 induction) only show marginal negative selection (*SI Appendix*, Fig. S6 *B* and *C*).

### Genome-Wide CRISPR Screen Targeting CTCF Binding Sites.

**Fig. 3.**
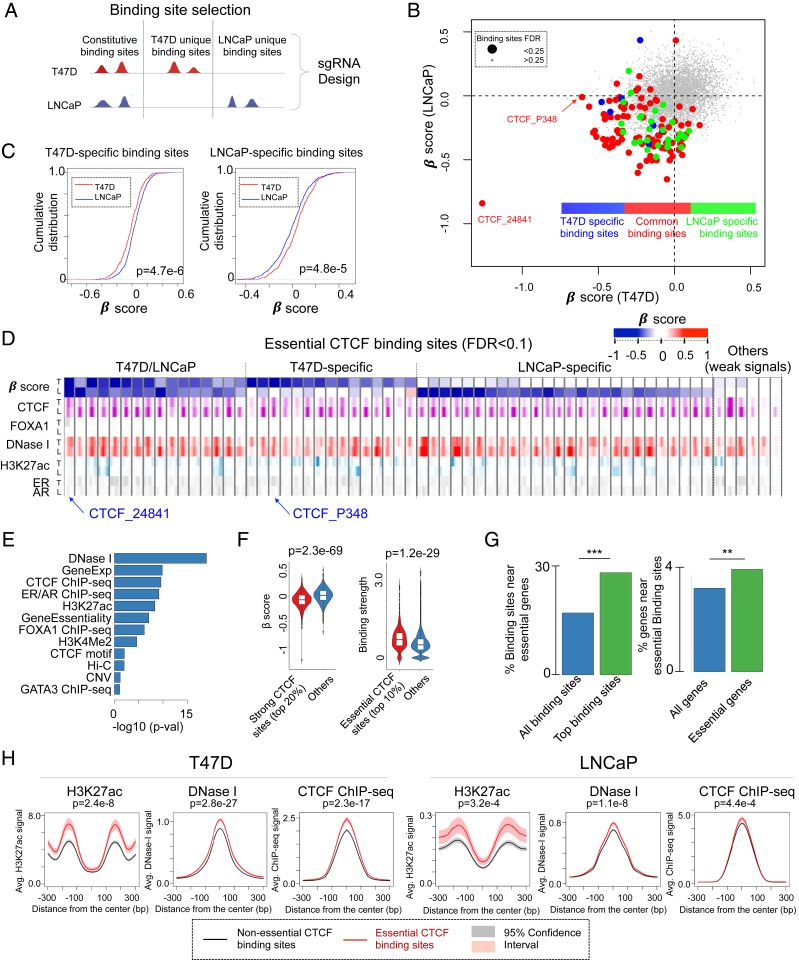
Genome-wide CRISPR screens for CTCF binding sites. (*A*) CTCF binding site selection procedure in screening library design. (*B*) The β-scores of all CTCF binding sites in T47D and LNCaP cells. Binding sites are colored by their appearances in both cell lines: Binding sites that only appear in T47D (or LNCaP) are colored in blue (or green), while common binding sites are colored in red, respectively. (*C*) The cumulative distribution of β-scores of T47D cell-specific and LNCaP cell-specific CTCF binding sites in T47D (red) and LNCaP (blue) cells. The *P* values are calculated by the Kolmogorov–Smirnov test. (*D*) The chromatin features of selected binding sites with statistical significance coming out of the cistrome screens. (*E*) The rankings of all features associated with the functions of CTCF binding sites. For each feature, we compare its signal distribution between the top 5% essential binding sites vs. other binding sites. The average of *P* values (calculated using the Mann–Whitney *U* test) across 2 cell lines is used to measure the relevance of each feature. (*F*) The β-score distribution of the strongest CTCF binding sites vs. others, and the binding strength of the top essential CTCF binding sites vs. other binding sites in T47D cells. (*G*) The percentage of all (and essential) CTCF binding sites that are within 100 kb of essential genes in T47D cells, and the percentage of all (and essential) genes near essential CTCF binding sites. Essential genes are genes with the 10% lowest β-scores from genome-wide CRISPR gene screens in T47D cells. **P < 0.01, ***P < 0.001. (*H*) The epigenetic features of essential binding sites vs. nonessential binding sites.

We next studied the essentiality of the binding sites of CTCF, a chromatin structure regulator. Although CTCF was not identified as a top essential gene in T47D or LNCaP cell lines in the gene screens using a stringent FDR cutoff of <0.05, loss of CTCF is negatively selected in the screens using a higher FDR (FDR = 0.32 in LNCaP). We have validated this fitness defect (30) upon CTCF knockout in T47D cells (*SI Appendix*, Fig. S7 *A*–*C*). We designed CRISPR/Cas9 knockout screening libraries targeting 2 types of CTCF binding sites, constitutive CTCF binding sites that are shared across multiple cell types, as well as sites that are specific to T47D and LNCaP cells (Fig. 3*A*, Table 1, and *SI Appendix*, Fig. S7*D*). The sgRNA design and the screening procedure are similar to FOXA1 cistrome screens, as previously described (*SI Appendix*, Table S4). Overall, the screening results in both cell lines are of high quality and the MAGeCK-VISPR copy-number correction module reduced the effects of genomic copy-number variations (*SI Appendix*, Fig. S7 *E*–*G* and Table S5). There are 150 CTCF binding sites that are essential in at least 1 cell line with statistical significance (FDR < 0.25; Fig. 3*B* and *SI Appendix*, Fig. S8*A*). CTCF binding sites that are specific to T47D or LNCaP cells tend to have lower β-scores in the corresponding cell types (Fig. 3 *B* and *C*), indicating a putative cell type-specific role for these binding sites. Possible features (*SI Appendix*, Fig. S8*B*) associated with the essentiality of CTCF binding sites include open chromatin (DNase I signal), nearby gene expression and essentiality (*SI Appendix*, Fig. S8*C*), as well as chromatin modification and lineage-specific TF binding (H3K27ac, ER/AR ChIP-seq signals) (Fig. 3 *D* and *E*). Interestingly, CTCF binding strength is strongly associated with binding site essentiality (Fig. 3*F*), in contrast to FOXA1 binding (Figs. 1*F* and 2*G*).

**Fig. 4.**
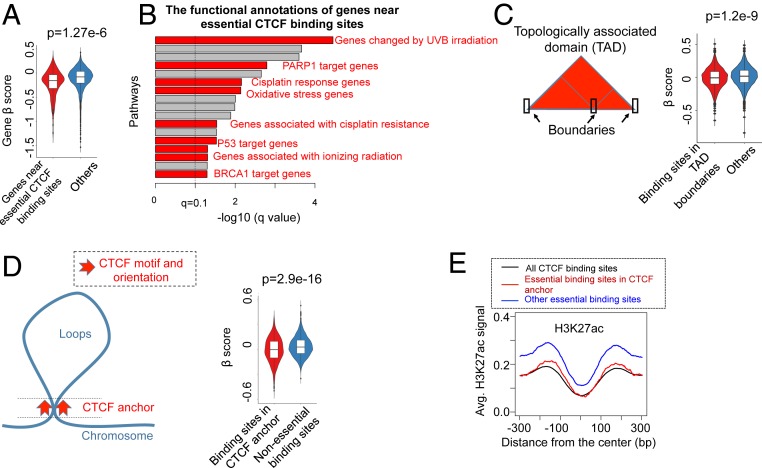
Essential CTCF binding sites display distinct types of CTCF binding. (*A*) The β-score distribution of genes near essential CTCF binding sites, compared with all genes in the genome. The *P* value is calculated using the Mann–Whitney *U* test. (*B*) The functional analysis of genes near essential CTCF binding sites using the GREAT prediction tool (46). Enriched terms related to DNA damage and stress response are highlighted in red. (*C*) The β-score distribution of binding sites in the boundaries of TADs. (*D*) The β-score distribution of binding sites in CTCF anchors, or regions that contact chromosome loops and with CTCF motifs that are head-to-head oriented. The anchor annotation is extracted from Hi-C experiments (31). (*E*) The H3K27ac signal strength distribution of essential binding sites in CTCF anchors, other essential binding sites, and all CTCF binding sites.

CTCF may serve as a typical transcription factor to regulate target gene expression or an insulator that defines boundaries in chromatin structure. Similar to the transcription factor FOXA1, essential CTCF binding sites tend to be closer to essential genes (Fig. 3*G*) and have higher levels of H3K27ac (Fig. 3*H*) and ER/AR signals (*SI Appendix*, Fig. S8*D*). Genes near essential CTCF binding sites tend to be more essential (Fig. 4*A*), and their functions are enriched in DNA damage response pathways (e.g., UVB irradiation, radiation, and cisplatin and P53/BRCA/PARP1 function; Fig. 4*B*). However, essential CTCF binding sites generally have weaker H3K27ac signals compared with FOXA1 binding sites (Figs. 2*A* and 3*D*), implying that they do not all function as canonical enhancers. We next examined CTCF binding sites in the boundaries of topologically associated domains (TADs) or in CTCF anchors—the contact regions of chromosome loops [extracted from Hi-C data (31)] that contain 2 head to head-oriented CTCF motifs (Fig. 4 *C* and *D*). Both TADs and anchors are critical to chromosome loop formation. We found CTCF binding sites in both regions tend to be more essential than others (Fig. 4 *C* and *D*), confirming the critical roles of both regions. Furthermore, essential binding sites in CTCF anchors tend to have weaker H3K27ac signals compared with essential binding sites not in the anchors (Fig. 4*E*), indicating that these binding sites function in ways distinct from essential enhancers. To further study the functional consequences of disrupting this type of CTCF binding site, we chose an essential CTCF binding site (CTCF_P348), located at the boundary between 2 TADs, for further functional validation (*SI Appendix*, Fig. S9*A*). Individual sgRNAs induced a high percentage of indels in the corresponding binding sites (*SI Appendix*, Fig. S9 *C* and *D*), and the CRISPR/Cas9–mediated mutagenesis of the CTCF_P348 binding site by 2 independent sgRNAs decreased the cell proliferation in the competitive growth assay (*SI Appendix*, Fig. S9*E*). Disruption of CTCF_P348 by the individual sgRNA-mediated CRISPR/Cas9 targeting compromises the binding of CTCF to these sites (*SI Appendix*, Fig. S9*F*).

### Essential Cistrome Modeling and Validation by pgRNA and Disease Association.

**Fig. 5.**
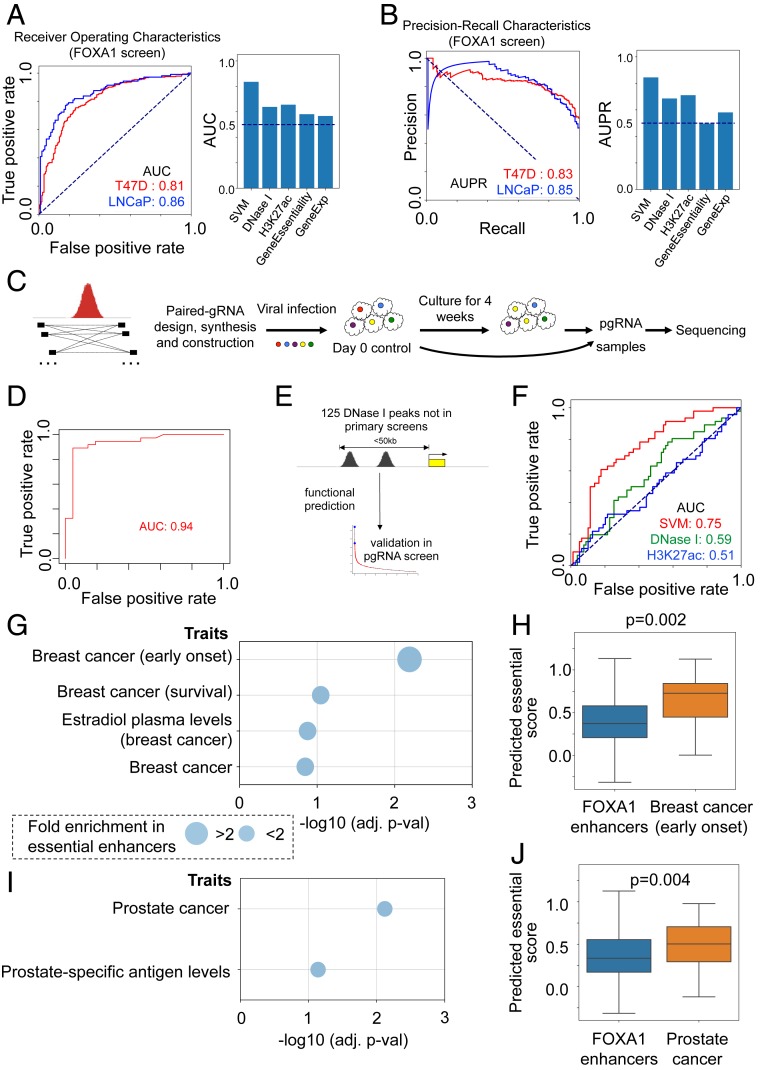
Predicting and validating essential enhancers. (*A*) The receiver operator characteristic (ROC) curves of different approaches for predicting FOXA1 binding site essentialities using different combinations of features. The AUC values using the SVM and individual features are also shown. (*B*) The precision-recall characteristic (PR) curves of *A*. The area under the PR curve (AUPR) of different approaches is shown. (*C*) An overview of the design of the pgRNA library and the screening strategy. Up to 25 pgRNAs are designed to knock out each binding site. (*D*) The ROC curve of the primary screening results in pgRNA screens. (*E*) The validation procedure of the prediction model using 125 DNase I binding sites that are not included in the sgRNA screening library. (*F*) The ROC curve of the prediction model (and 2 single features) in predicting the functions of 125 DNase I binding sites. (*G*) The enrichment of breast cancer-related variants over essential enhancers in T47D cells. The adjusted *P* values (using the χ^2^ test) are shown. Circle sizes indicate the fold enrichment over essential enhancers (>2 or <2). (*H*) The predicted score distribution of all FOXA1-bounded enhancers, and enhancers that carry variants of “breast cancer (early onset).” The *P* value is calculated using the Wilcoxon rank-sum test. (*I* and *J*) The same analysis of *G* and *H* in LNCaP cells.

To systematically evaluate both FOXA1 and CTCF screening results and associated features, we built a support vector machine (SVM)-based regression model to predict the essentiality of enhancers or cistrome binding sites, based on all associated features extracted from our results. We tested the performance of this model with the screening data using 5-fold cross-validations. Compared with using single features correlated with enhancer function to predict essentiality such as DNase I sensitivity or H3K27ac chromatin modification, our model performs significantly better with an area under the curve (AUC) of ∼0.8 (Fig. 5 *A* and *B* and *SI Appendix*, Fig. S10 *A* and *B*). We further investigated the binding sites for which the SVM model made the incorrect prediction, including essential binding sites that were predicted as nonessential (false negatives; FNs), or nonessential binding sites that were predicted to be essential (false positives; FPs). We found that FP binding sites tend to have stronger DNase I sensitivity and H3K27ac marks (similar to essential binding sites), while FN binding sites harbor lower levels of such signals (similar to nonessential binding sites; *SI Appendix*, Fig. S10*C*). This indicates that some nonessential binding sites that have strong epigenetic characteristics of functional enhancers may have functions that are not related to cell growth or may be redundant with other enhancers in the same gene. On the other hand, some essential binding sites may not have typical epigenetic signatures, consistent with the existence of active enhancers that do not bear active epigenetic marks (9).

We further evaluated the results of our CTCF/FOXA1 primary screens using a pgRNA CRISPR screening technology as a validation screen (7). A focused pgRNA library was constructed by targeting DNase I-accessible regions that are close to the essential genes in T47D cells, as well as top binding sites in the primary CTCF/FOXA1 screens (Table 2 and *SI Appendix*, Table S7). Up to 25 pgRNAs flanking the binding sites or putative enhancers were designed (Fig. 5*C*). As positive controls, pgRNAs targeting the exons or promoters of genes whose loss suppresses (or promotes) cell growth were included. We screened T47D cells grown in full medium with 2 biological replicates and analyzed the results using MAGeCK-VISPR (26) (*SI Appendix*, Table S8). As expected, the promoters and exons of essential genes (e.g., *ESR1*, *FOXA1*, and *RPS9*) are strongly negatively selected (*SI Appendix*, Fig. S10 *D* and *E*), indicating the reliability of the pgRNA screens.

**Table 2.** A summary of the secondary paired-guide RNA screening library

	Binding sites	Paired-guide RNAs
Enhancers near negatively selected genes (ESR1, FOXA1, GATA3, MYC)	92	2,291 (25 pairs per binding site)
Enhancers near positively selected genes (PTEN, TSC1, RB1, CSK)	46	1,150 (25 pairs per binding site)
Selected hits in CTCF/FOXA1 screens	58	1,450 (25 pairs per binding site)
Promoters of the selected genes	N.A.	259 (∼25 pairs per promoter)
Positive control (pairs targeting AAVS1 loci and the exons of essential genes)	146 genes	730 (5 pairs per gene)
Negative control (pairs targeting AAVS1 loci)	N.A.	400

N.A., not applicable.

There are in total 80 binding sites that are included in both primary and secondary screens. Among them, we compared binding sites that are selected with statistical significance (FDR < 0.25) by both screens (*SI Appendix*, Fig. S10*F*). Of the 13 essential binding sites that are identified as statistically significant in primary screens, 70% (9/13) were confirmed to be essential (with FDR < 0.25) in the pgRNA screens, demonstrating the high specificity of the primary screening results. There were 25 binding sites in the primary screen with FDR >0.25 that were statistically significant hits in the validation screen, indicating that screens using the pgRNA may be more sensitive in identifying essential binding sites. For example, CTCF_69346 (CTCF_P346), a CTCF binding site that is 6 kb away from CTCF_P348, was not negatively selected in the primary screen (FDR = 1.0), but was essential in the pgRNA screen (FDR = 0.002; *SI Appendix*, Fig. S10*E*). Overall, pgRNA screening validated the top hits in primary screens with high accuracy, with an AUC close to 0.94 (Fig. 5*D*).

We further evaluated the performance of our predictive model, using the 125 DNase-seq peaks in the pgRNA library not in the sgRNA libraries used for the primary screens (Fig. 5*E*). We predicted the essentiality of these 125 DNase-seq peaks using the model trained on FOXA1 primary screening data, and compared them with the experimental results in the pgRNA screen. The overall AUC approaches 0.75, demonstrating the high performance of our prediction model (Fig. 5*F*).

Finally, we used our predictive model to evaluate the essentiality of enhancers that overlap with disease-associated single-nucleotide polymorphisms (SNPs) that are identified from genome-wide association studies (GWAS). We focused only on noncoding SNPs associated with breast cancer in T47D cells and prostate cancer-associated SNPs in LNCaP cells. Among the 22 breast cancer-related traits, 4 are enriched in predicted essential enhancers with statistical significance (FDR < 20%; Fig. 5 *G* and *H*), and “breast cancer (early onset)” is the top trait, followed by “breast cancer (survival).” Enhancers bearing these SNPs have higher predicted essential scores compared with random DNase I-hypersensitive sites or ER or FOXA1 binding sites (*SI Appendix*, Fig. S10*G*). These enhancers are also proximal to coding genes that are up-regulated in luminal breast cancer (*SI Appendix*, Fig. S10*H*). Similarly, 2 out of 8 prostate cancer-associated traits are significantly enriched in essential enhancers, and the strongest enrichment comes from the “prostate cancer” trait itself (Fig. 5 *I* and *J*). Therefore, our predictive model can be used to infer the functions of GWAS-associated SNPs that affect cell fitness.

## Discussion

Dissecting the functions of putative *cis*-regulatory elements in mammalian cells has been challenging. We have established a CRISPR/sgRNA–based screening approach to investigate the essentiality of the binding sites of 2 important transcription factors, FOXA1 and CTCF. We validated the essential roles of top hits and the regulation of their target genes using experimental approaches including CRISPR/pgRNA screening. Based on the screening data, we further evaluated genomic and epigenomic features associated with essential enhancers, and built machine-learning models that predict the functions of sites that are not included in the screen. The model can also be used to explain disease-associated SNPs that affect cell growth.

Our study demonstrated genome-wide cistrome screens as a promising technology to characterize the functions of transcription factor binding in detail. By comparing the essential FOXA1 and CTCF cistromes, we found that essential binding sites and essential genes tend to be close to each other for both the interrogated FOXA1 and CTCF cistromes. Although a minor subset of interrogated FOXA1 binding sites (18.3%) was chosen based on their proximity (<50 kb) to essential genes, the remaining FOXA1 sites and all of the CTCF binding sites were chosen independent of this proximity criterion. These results indicate a general link between essential binding sites and the essential coding regions nearby. We further identified distinct features associated with the essentiality of FOXA1 and CTCF binding sites. FOXA1 binding sites bear the hallmarks of canonical enhancers, evidenced by strong DNase I and H3K27ac signals, and their functions can be predicted from nearby gene expression and essentiality. In contrast, CTCF essential binding sites fall into 2 classes: They may be either transcriptional enhancers or critical elements in chromosome organization. Binding sites of the first type have characteristics similar to FOXA1 binding sites, while the second class of sites are distinct. Interestingly, CTCF binding strength predicts the essentiality of a binding site, while no association is found between the strength of FOXA1 binding to a site and its essentiality. This may be because FOXA1 works together with other transcription factors such as ER and AR to regulate gene expression, and thus the binding strength of 1 single transcription factor may not capture the important features of combinatorial transcription factor binding. These results also highlight that modeling the functionality of the binding sites of different classes of transcription factors will require additional functional screens.

Several computational methods are available to predict active enhancers from genetic and epigenetic features (32–36) or from the screening experiments measuring the expression of a gene (9, 11). It is still tremendously challenging to systematically assign many enhancers to their bona fide target genes or particular phenotypes experimentally. A recent study identified hundreds of enhancer–gene pairs using a high-throughput CRISPR inactivation (dCas9-KRAB) approach to perturb enhancers followed by single-cell RNA sequencing (37). Here our approach investigated over 10,000 putative enhancers downstream of 2 essential transacting factors and linked them to a specific cellular function or phenotype (essentiality or fitness). We generated genome-scale experimental data of *cis*-acting element perturbations, and constructed a machine-learning model based on these experimental data to predict functional binding sites. We applied this strategy to identify the functional *cis*-elements that are critical to breast and prostate cancer cell growth. In contrast, previous computational methods cannot predict the possible phenotypes that each enhancer is involved with, or are based on the data from a limited number of enhancers proximal to the target gene. Our model may also be helpful to prioritize the functional or important binding sites from other ChIP-seq data. In addition, our experimental and computational framework here can be extended to study the function of *cis*-regulatory elements in other contexts.

CRISPR/Cas9 screens for noncoding elements can be based on either sgRNA (6, 10–13) or pgRNA (7, 9, 38, 39) strategies. In our study, we performed primary sgRNA-based screening and validated the results using a pgRNA approach. We found that pgRNA screening not only confirmed top hits found in sgRNA-based screening but also identified additional hits that were not found by sgRNA screens. The combinatorial use of both sgRNA and pgRNA approaches could be very helpful to reduce the false positives as introduced by the potentially off-target effects of low-specificity guides in sgRNA strategies (40).

There are potential limitations to our current studies. First, our screening approach only selects binding sites that affect cell growth, and does not identify enhancers that play other functional roles (e.g., differentiation). Large-scale enhancer screens for phenotypes other than cell growth (e.g., protein expression that can be selected through FACS sorting) will enable functional studies of enhancers of a variety of functions. Second, genomic deletion of putative *cis*-elements may also alter other functional elements such as long noncoding RNAs, which may contribute to the screening outcome as well. A refined sgRNA design based on these results may be possible to reduce overlap with other annotated genomic features. The use of orthogonal validation approaches may also reduce the number of false positives. Third, systematic library bias (mainly due to differential sgRNA efficiency for each targeting element or gene for each library) is inevitable for all current CRISPR screening studies, which may also necessitate the use of independent approaches or repeated studies employing a variety of libraries to fully capture the essential cistrome. In addition, some studies have reported the preferential binding of Cas9 to open chromatin regions (41). To reduce the biases of Cas9 binding, we used sgRNAs targeting strong FOXA1 binding sites as negative controls in the FOXA1 cistrome screening study. However, in the future it will be desirable to further model and correct for the binding preferences of Cas9, by designing negative controls in nonfunctional, open chromatin regions, and by considering the effect of open chromatin in the analysis.

In summary, we have demonstrated the feasibility of screening for the function of large numbers of *cis*-regulatory elements in a pooled format using a CRISPR/Cas9 sgRNA and pgRNA approach. For lineage-selective enhancer-binding transcription factors such as FOXA1, we have developed a model based on epigenomic features that is predictive of the essential function of a subset of the transcription factor binding sites for cell growth in culture. Importantly, the sites predicted by this model significantly overlap with germline variants associated with cancer risk and progression identified by GWAS, demonstrating that the features selected for the model by the CRISPR/Cas9 sgRNA screening results are clinically relevant.

## Materials and Methods

Detailed description of materials and methods can be found in *SI Appendix*, *Methods*.

### Cell Culture and Reagents.

Breast cancer T47D cells were maintained in Dulbecco’s modified Eagle’s medium (DMEM) supplemented with 10% fetal bovine serum (FBS) as the full medium condition. When stimulated with the estrogen 17β-estradiol, T47D cells were cultured in phenol red-free DMEM with 10% charcoal/dextran–treated FBS for at least 3 d after switching from the full media. Prostate cancer LNCaP cells were cultured in RPMI 1640 media supplemented with 10% FBS as the full medium condition. HEK293FT cells were grown in DMEM with 10% FBS. The antibodies were purchased from the following companies: GAPDH (FL-335; Santa Cruz; sc-25778), ERα (HC-20; Santa Cruz; sc-543), FOXA1 (Abcam; ab23738), and CTCF (EMD Millipore; 07-729).

### CRISPR Library Synthesis and Construction.

The pooled synthesized oligos were PCR-amplified and then cloned into the lentiCRISPRv2-puro vector via the *Bsm*BI site by Gibson assembly. The ligated Gibson assembly mix was transformed into self-prepared electrocompetent DH5α *Escherichia coli* by electrotransformation to reach efficiency with at least 20× coverage representation of each clone in the designed library. The transformed bacteria were cultured directly in liquid LB medium for 16 to 20 h at low temperature (16 °C) to minimize the recombination events in *E. coli*. The library plasmids were then extracted with the GenElute HP Endotoxin-Free Plasmid Maxiprep Kit (Sigma; NA0410-1KT). To confirm the designed guide RNA sequences were successfully cloned into the plasmid library, we PCR-amplified the guide RNA sequences, prepared sequencing libraries, and employed the NextSeq 500 sequencing platform to validate the inserted gRNA sequences as a stringent QC for the plasmid library. After alignment to our designed sequences, more than 99.92% of designed gRNA sequences were present in our plasmid libraries, indicating the high quality of the libraries.

### Pooled Genome-Wide CRISPR Screen.

FOXA1 and CTCF cistrome-targeting plasmid libraries under the lentiviral lentiCRISRPv2-puro backbone were first transfected along with pCMV8.74 and pMD2.G packaging plasmids into HEK293FT cells using X-tremeGENE HP DNA Transfection Reagent (Roche; 6366236001) to generate a CRISPR component-expressing lentivirus. Virus-containing media were harvested at 48 and 72 h posttransfection, and the media were spun down at 1,000 rpm for 5 min to remove floating cells and cell debris. The virus supernatant was carefully collected, aliquotted, and stored at −80 °C for further use. The virus titer and MOI (multiplicity of infection) were tested before proceeding to the genome-wide screen.

For the full medium screen, 1 × 10^8^ to 2 × 10^8^ T47D or LNCaP cells were infected with CTCF or FOXA1 cistrome-targeting lentiviral libraries with MOI ∼0.3. Two days later, the infected cells were selected with puromycin (3.5 μg/mL for T47D cells and 1.5 μg/mL for LNCaP cells) for 3 d to get rid of any noninfected cells before changing back to normal media. After 2 d of recovery post puromycin selection, around a half portion of cells (at least 3 × 10^7^ cells, ∼300× coverage for each library) was collected as the day 0 sample and stored at −80 °C for later genomic DNA isolation. The remaining half of cells were continually cultured until 4 wk later before harvesting as the end-point sample. For screens in T47D cells under vehicle and E2 condition, cells were cultured in either vehicle (ethanol) or 10 nM E2-containing white medium for an additional 5 wk after harvesting the day 0 sample. Genomic DNA from day 0 and end-time point samples was extracted.

Gene screens in T47D and LNCaP cells cultured under full medium were performed similar to the cistrome screens. The sgRNA library for gene screens targeting ∼18,000 genes in the human genome was designed by our laboratory with an up-to-date algorithm to improve the specificity and efficacy of gRNAs and is described in our recent studies (42). Samples of day 0 and day 21 were used to quantify the gRNA abundance with a similar library preparation protocol as the cistrome screens. The data were analyzed by MAGeCK-VISPR (26).

### Genetic and Epigenetic Features Associated with Screening Outcomes.

We collected a set of genetic and epigenetic features in T47D and LNCaP. The H3K27ac and RNA-seq data were extracted from our previous studies (43). Other ChIP-seq data were extracted from our cistrome database (cistrome.org), and only datasets that passed the quality control measurements in the database were used for downstream analysis. For each putative enhancer identified by DNase I, the normalized ChIP-seq signals of the 150-bp window (centered on the DNase I peak summit) were collected as features. For histone modification ChIP-seq data, the window size was extended to 300 bp.

### Predicting Enhancer Functions.

For building machine-learning models to predict enhancer functions, we use both essential and nonessential sites in the screening as training samples. Since the number of significant sites is few, we increased the threshold (negative rank < 300) to select more (but less statistically significant) sites as essential sites. Nonessential sites were chosen such that they were neither negatively nor positively selected (*P* > 0.5), and their absolute log fold change was less than 0.1. In all of the datasets, essential and nonessential sites were balanced (essential-to-nonessential rate is between 0.85 and 1.1).

The SVM toolkit in the scikit-learn package (https://scikit-learn.org) was used for training and prediction. We used a genetic algorithm combined with SVM (GA-SVM) to select best feature combinations (44, 45). Briefly, GA-SVM is an iterative process, where a set of feature combinations are subjected to randomly adding/removing/changing 1 feature at each iteration. Features that reach better prediction performance have a higher chance of going to the next iteration. This process is repeated several times to select the best combination of features. The entire dataset was split into the training and validation sets, where the training dataset was used to train the SVM, and the area under the receiver operator characteristic value calculated on the validation set was used to evaluate feature combinations.

GWAS-associated SNPs and their traits were downloaded from the GWAS Catalog (https://www.ebi.ac.uk/gwas/). If the location of the SNP overlaps with a known DNase I peak in T47D or LNCaP, the corresponding DNase I binding site serves as the SNP-bearing enhancer. If no DNase I peak overlaps with the SNP location, we then search for possible FOXA1, ER, or GATA3 binding sites. If none of these peaks overlaps with a SNP, we then consider a 150-bp window centered on that SNP as an “enhancer” for downstream analysis. The prediction algorithm was applied to evaluate whether these SNP-associated enhancers are essential.

## Supplementary Material

Supplementary File

Supplementary File

Supplementary File

Supplementary File

Supplementary File

Supplementary File

Supplementary File

Supplementary File

Supplementary File
