# Supplementary material for: Deciphering essential cistromes using genome-wide CRISPR screens
Source: Proc Natl Acad Sci U S A. 2019 Nov 14;116(50):25186–95. doi: 10.1073/pnas.1908155116 (PMC6911175; doi:10.1073/pnas.1908155116)
Supplement: Supplementary File [file pnas.1908155116.sapp.pdf]

Supporting Information

Deciphering Essential Cistromes Using Genome-wide CRISPR Screens

Teng Fei[1,2,3,#], Wei Li[3,4,#], Jingyu Peng[2,3,#], Tengfei Xiao[2,3], Chen-Hao Chen[3], Alexander Wu[3,5], Jialiang Huang[3], Chongzhi Zang[6], X. Shirley Liu[3,*], Myles Brown[2,*]

www.pnas.org/cgi/doi/10.1073/pnas.1908155\116

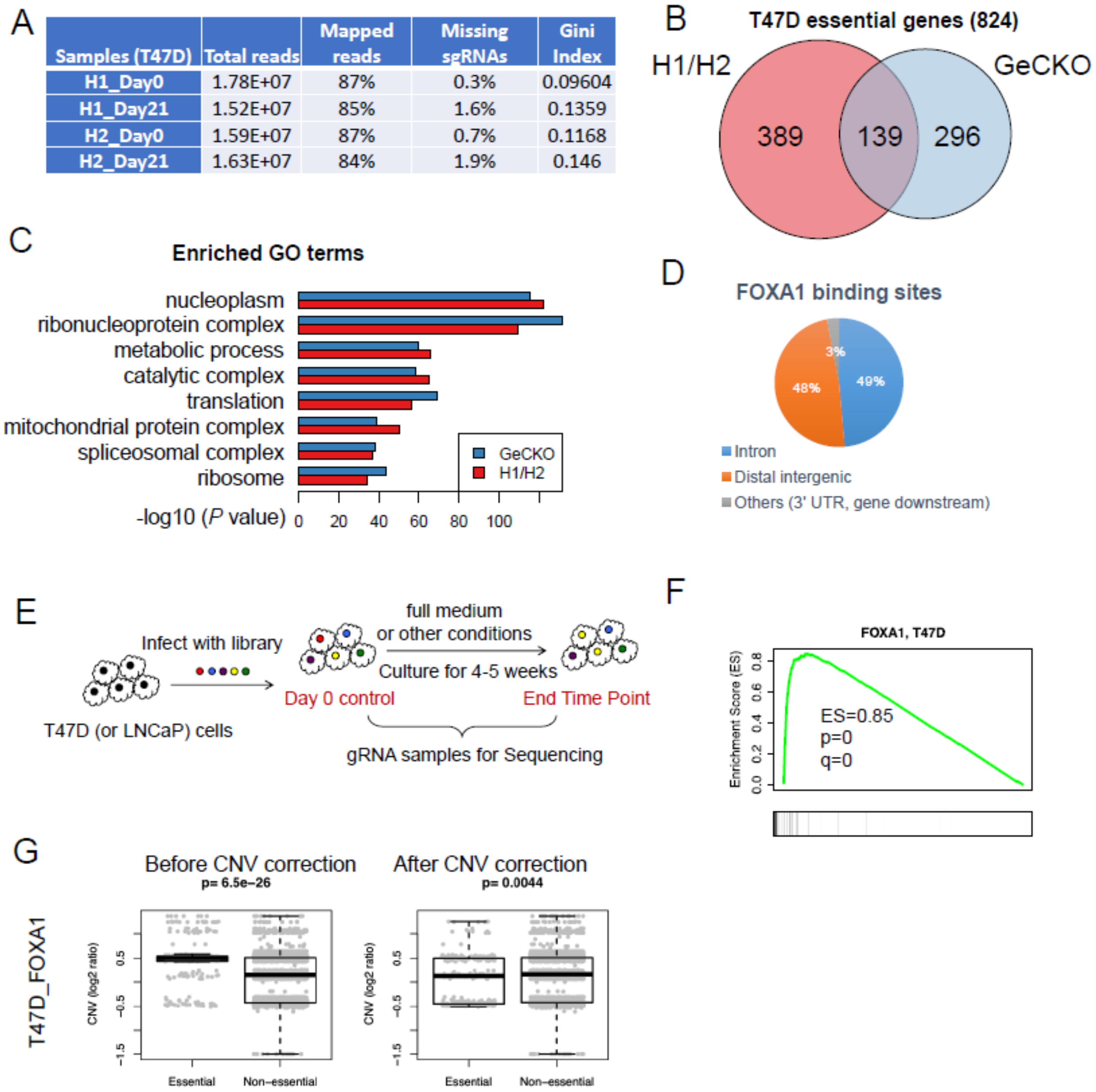

Figure S1. FOXA1 binding site screening.
(A-G) Genome-wide CRISPR gene screens in T47D cells. (A) Quality control (QC) measurements of gene screens on all samples, using the H1 or H2 gene libraries that we developed, at the beginning (Day0) or at the end (Day21) of the screen. (B) The overlap of T47D cell essential genes identified by our library or by GeCKO library (1). Note that the overlap rate is not very high, which is primarily due to the intrinsic library-specific biases (e.g. varied targeting efficiency of gRNAs in different libraries) but not an indicator of screen quality per se. (C) The enrichment of GO terms of essential genes, identified by the H1/H2 library or by the GeCKO library. (D) The distribution of designed

FOXA1 binding sites in different parts of the genome. (E) The screening procedure. (F) Gene Set Enrichment Analysis (GSEA) results of positive control genes (known essential genes) in negative selection ranked list of genes/binding sites of sgRNA cistrome screen. (G) MAGeCK CNV correction procedure reduces the biases of copy number variations in FOXA1 cistrome screens. The distribution of CNV of essential genes/binding sites (measured as log2 ratio) are shown before and after CNV correction procedure. Essential genes/binding sites are defined as 10% genes/binding sites that have the smallest β scores in each screen.

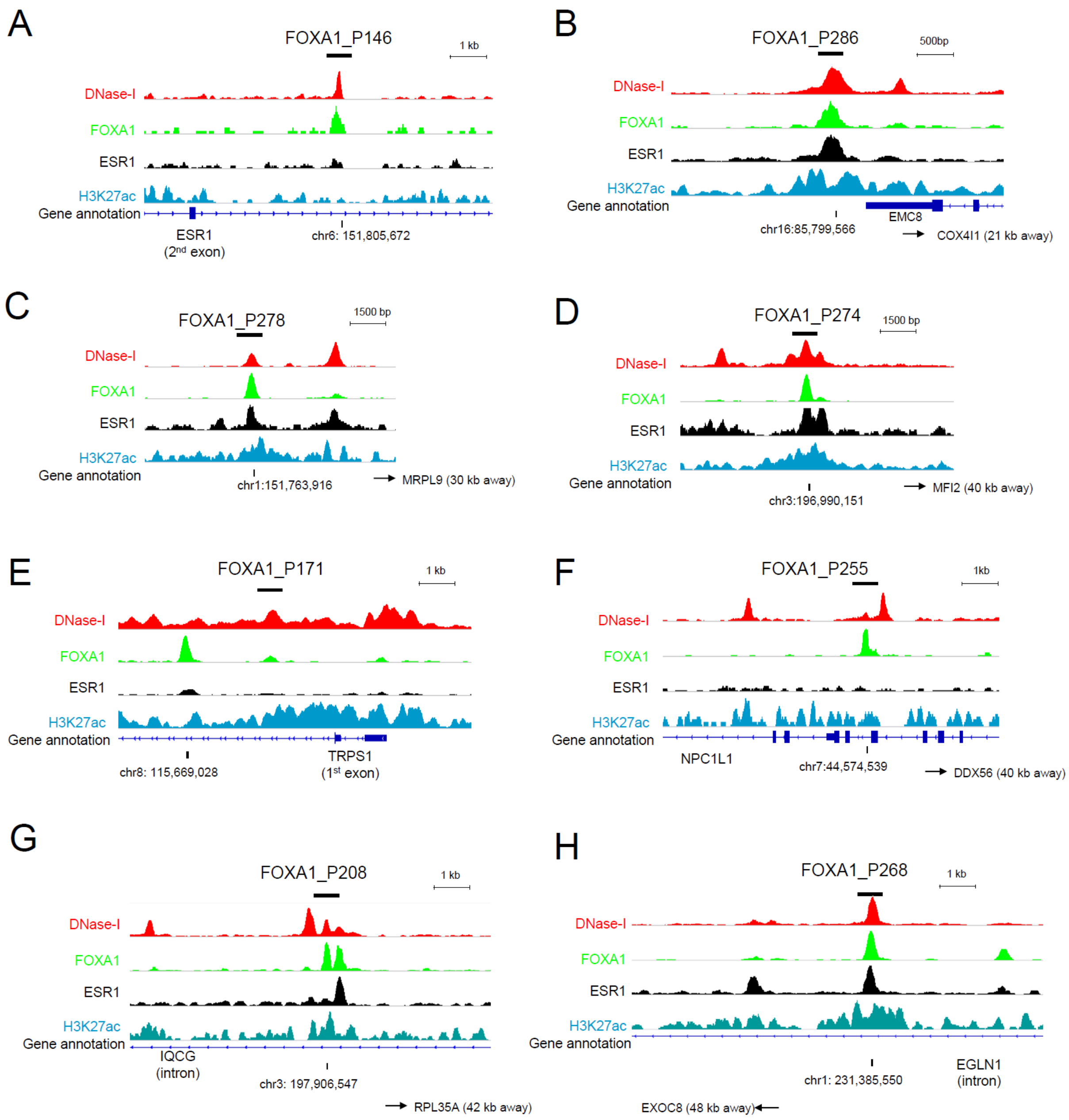

Figure S2. Top essential FOXA1 binding sites that are close to essential genes.

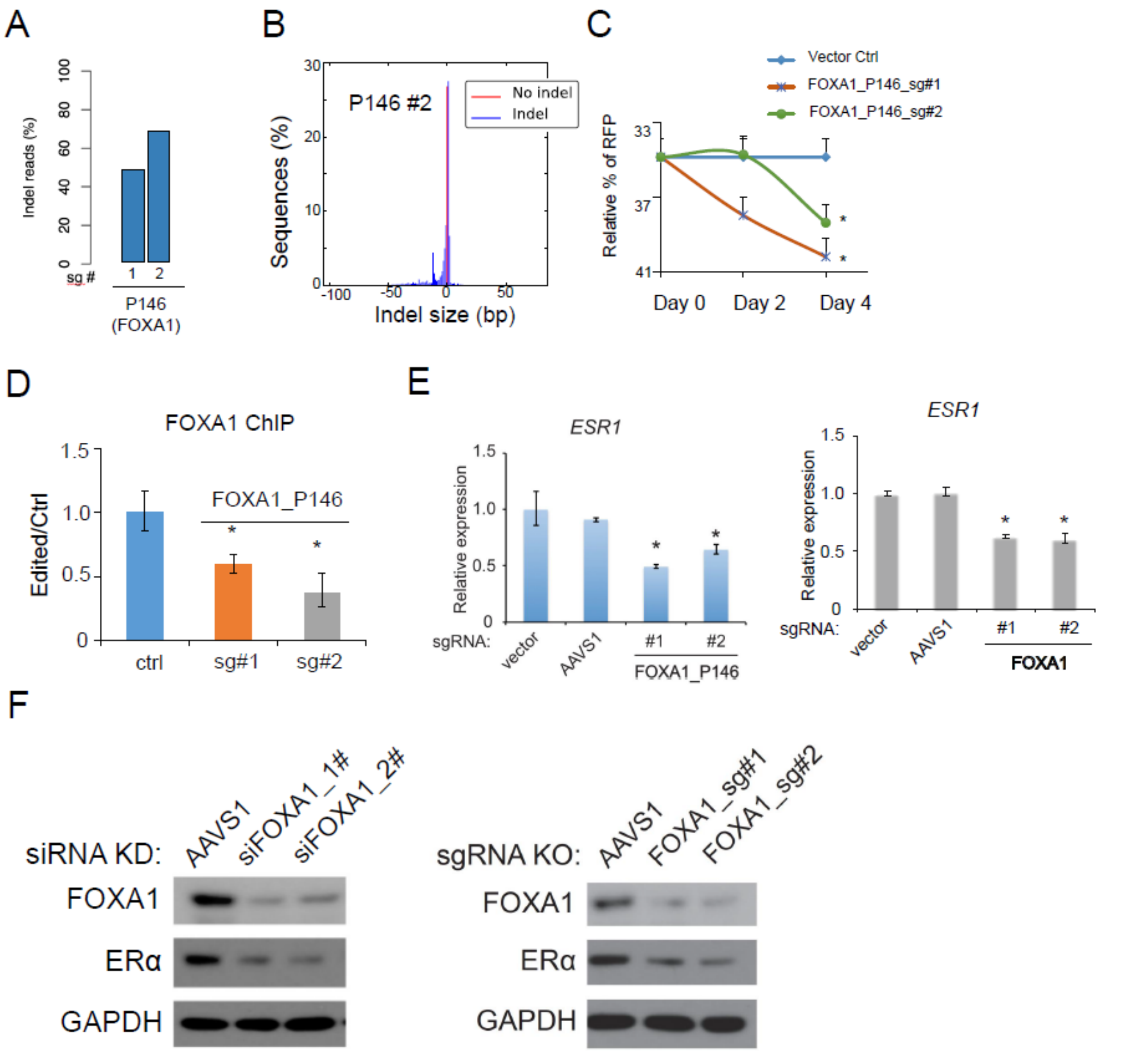

Figure S3. Experimental validation of top FOXA1 cistrome screen hits.
(A) The percentage of indel reads (from targeted DNA sequencing) from FOXA1_P146 loci using different sgRNAs. (B) The indel size distribution of knocking out FOXA1_P146 locus with sgRNA #2. (C) Competitive cell growth assay confirmed the cell growth effect after knocking out FOXA1_P146 using two different sgRNAs. CRISPR-targeted T47D cells with indicated sgRNAs or control were mixed with red fluorescence protein (RFP)-expressing non-edited cells with a ratio around 2:1 at Day 0 and continually cultured for 4 days. RFP fraction was determined at indicated time points by flow cytometry. The more essential of CRISPR-targeted site, the more percentage of RFP cells in the mixed population. **P*<0.05. (D) The relative FOXA1 binding on FOXA1_P146 after binding site knockout with two individual sgRNAs determined by ChIP-qPCR. **P*<0.05. (E) The relative mRNA expression of *ESR1* after knocking out the FOXA1_P146 binding site or

the *FOXA1* gene in T47D cells determined by qRT-PCR. Data were shown as mean ± SEM, n = 3, $^{*}P < 0.05$. (F) Perturbation of FOXA1 expression by either RNA interference knockdown (left) or CRISPR knockout (right) leads to reduced expression of ERα (*ESR1*-encoded protein) in T47D cells by Western blot analysis.

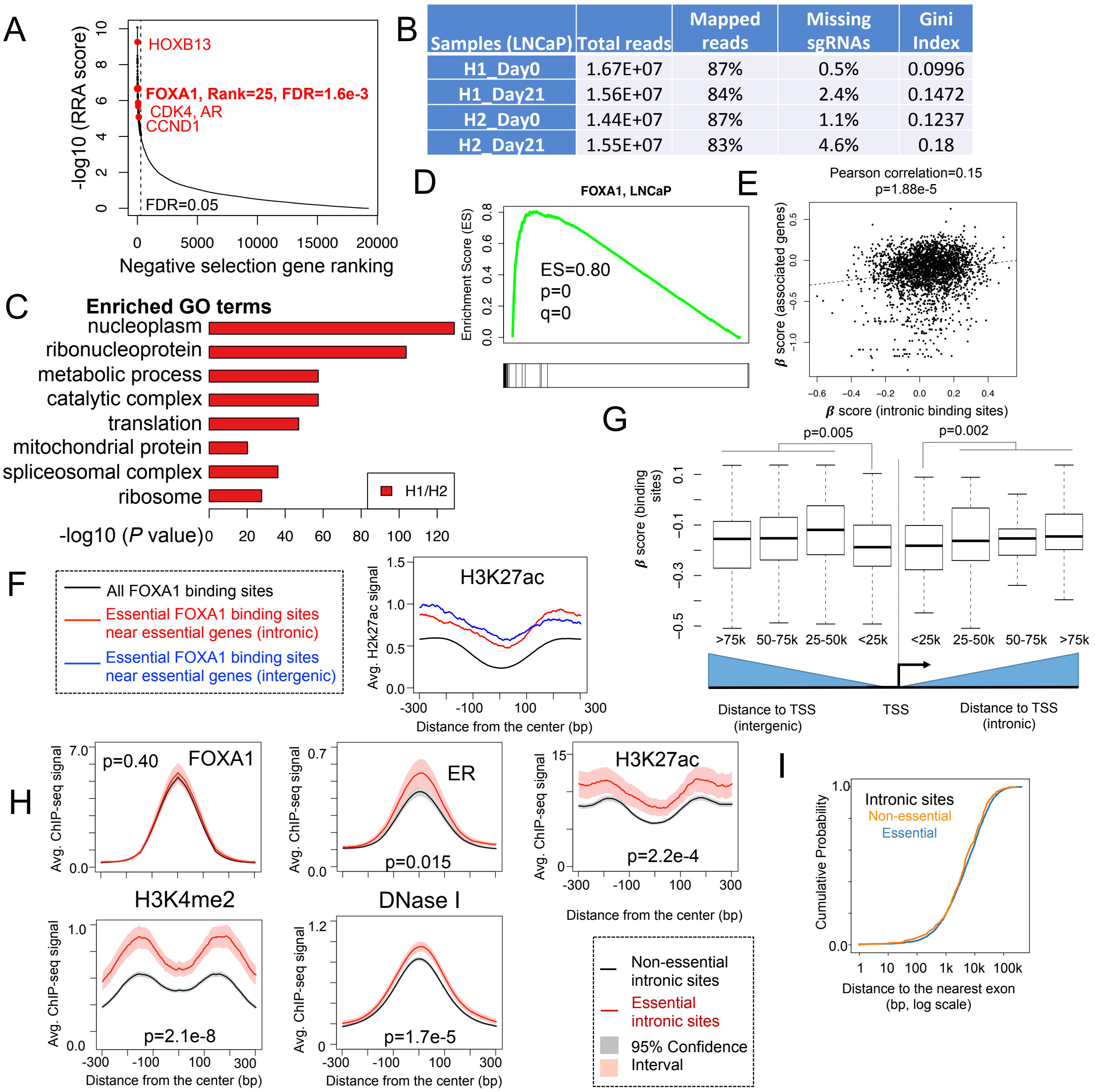

Figure S4. FOXA1 binding site screening in LNCaP cells and in introns.
(A) Genome-wide CRISPR screens for FOXA1 binding sites in LNCaP cells. (B) Quality control (QC) measurements of gene screenings on all samples, using H1 or H2 gene libraries we developed, in the beginning (Day0) or at the end (Day21) of the screen. (C) The enrichment of GO terms of essential genes in the gene screen. (D) Gene Set Enrichment Analysis (GSEA) results of positive control genes (known essential genes) in negative selection ranked list of genes/binding sites of sgRNA cistrome screen in LNCaP cells. (E) Pearson correlation of the β scores of intronic FOXA1 binding sites and the corresponding genes. (F) The H3K27ac signal strength of intronic and intergenic essential FOXA1 binding sites. (G) The distribution of β scores of FOXA1 binding sites, group by their relative position and distance to target gene TSS

(Transcription Start Site). (H) The ChIP-seq signals of ER, FOXA1, H3K4me2, H3K27ac, DNase I in intronic essential and intronic non-essential sites. (I) The distribution of distance to the nearest exon of intronic essential and non-essential sites.

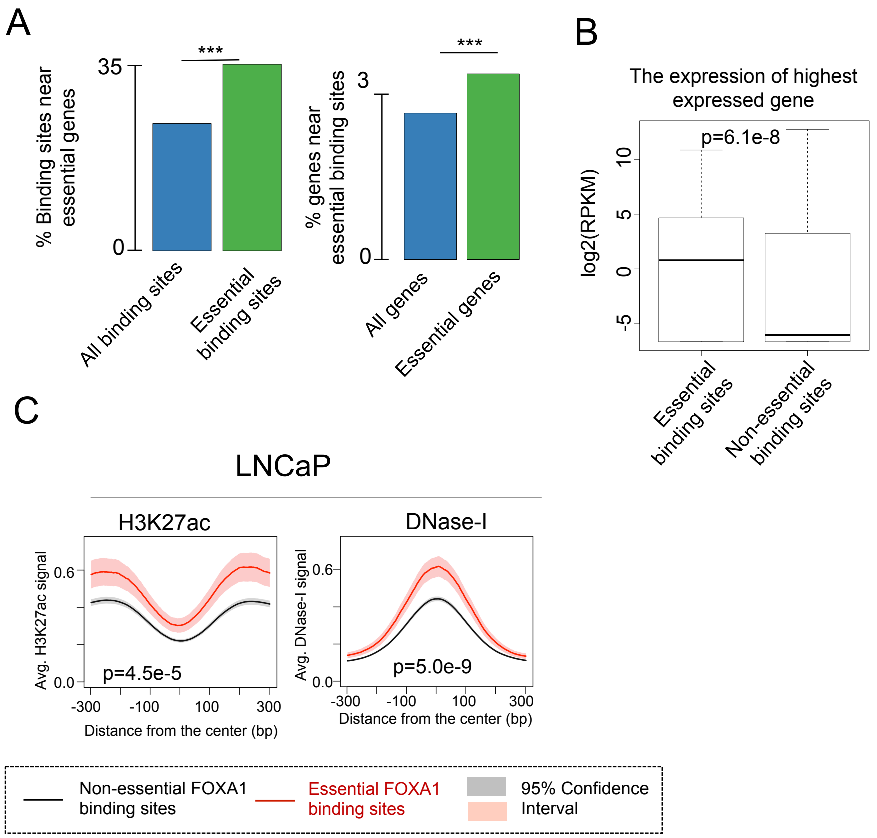

Figure S5. Features associated with FOXA1 binding site essentiality in LNCaP cells. (A) The percentage of all (and essential) FOXA1 binding sites that are within 100 kb of essential genes in LNCaP cells, and the percentage of all (and essential) genes near essential FOXA1 binding sites. Essential genes are genes with 10% lowest β scores from genome-wide CRISPR gene screens in LNCaP cells. ***$p<0.001$. (B) The expression of highest expressed genes near essential and non-essential FOXA1 binding sites. (C) The epigenetic features of essential FOXA1 binding sites vs. non-essential binding sites in LNCaP cells.

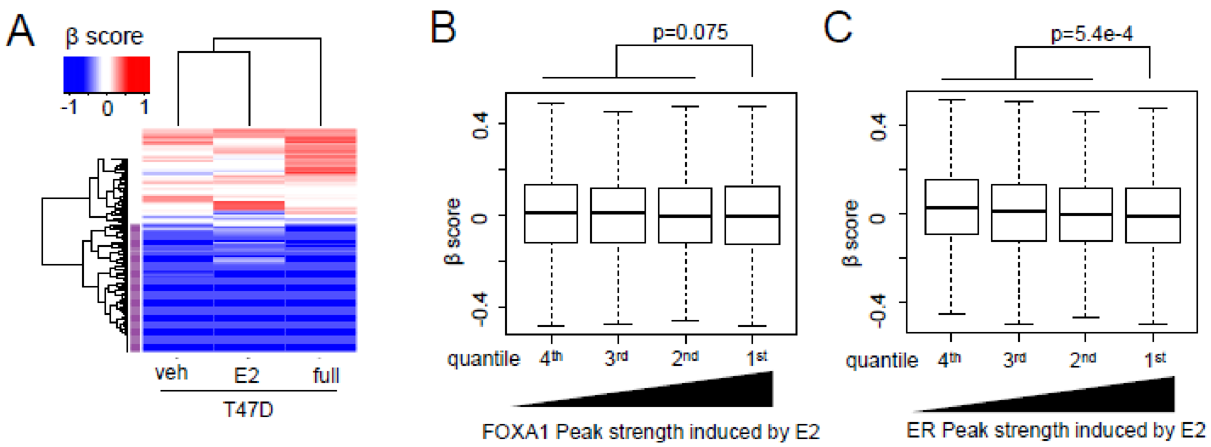

Figure S6. FOXA1 cistrome screen in T47D cells with different conditions.
(A) A hierarchical cluster of β scores for different culturing conditions in T47D cells. Only binding sites or genes with statistical significance are shown. Positive control essential genes are shown as purple bars in sidebar. (B-C) The distribution of the β scores of all FOXA1 binding sites in E2-treated T47D cells, grouped by their FOXA1 (B) or ESR1 (C) log-fold change of binding site strength in E2 vs. vehicle conditions.

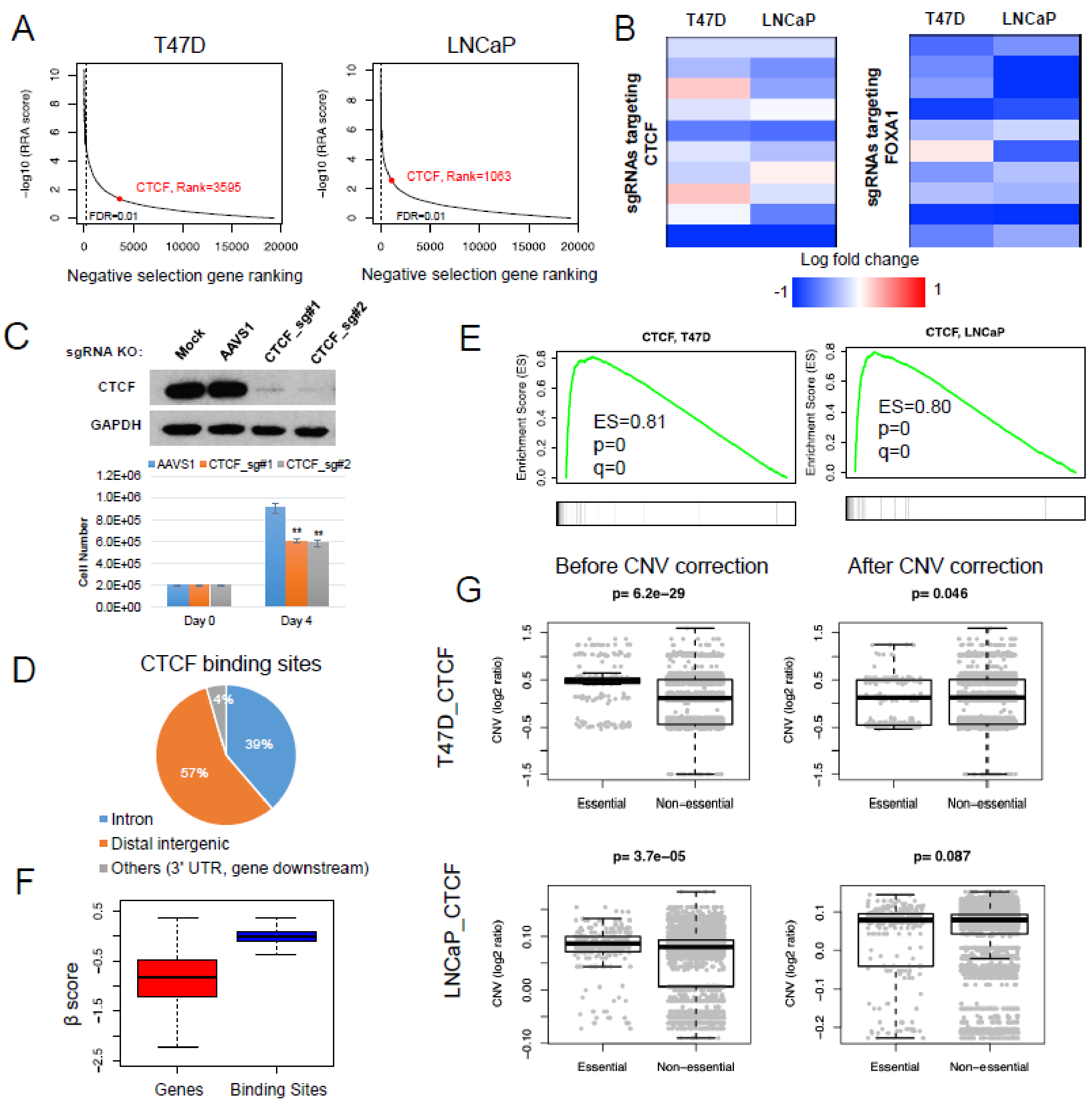

Figure S7. CTCF cistrome screen.
(A) The ranking of CTCF gene in essential gene list, measured by genome-wide CRISPR screens. (B) The log fold change of all sgRNAs targeting CTCF in both cell lines. (C) CTCF knockout by two different sgRNAs (upper) and cell growth assay (bottom). Data were shown as mean ± SD, n = 4, ***P* < 0.01. (D) The distribution of designed CTCF binding sites in different parts of the genome. (E) Gene Set Enrichment Analysis (GSEA) results of positive control genes (known essential genes) in negative selection ranked list of genes/binding sites of sgRNA cistrome screen. (F) The β score distribution of genes and binding sites. (G) MAGeCK CNV correction procedure reduces the biases of copy number variations in CTCF cistrome screens. The distribution of

CNV of essential genes/binding sites (measured as log2 ratio) are shown before and after CNV correction procedure. Essential genes/binding sites are defined as 10% genes/binding sites that have the smallest β scores in each screen.

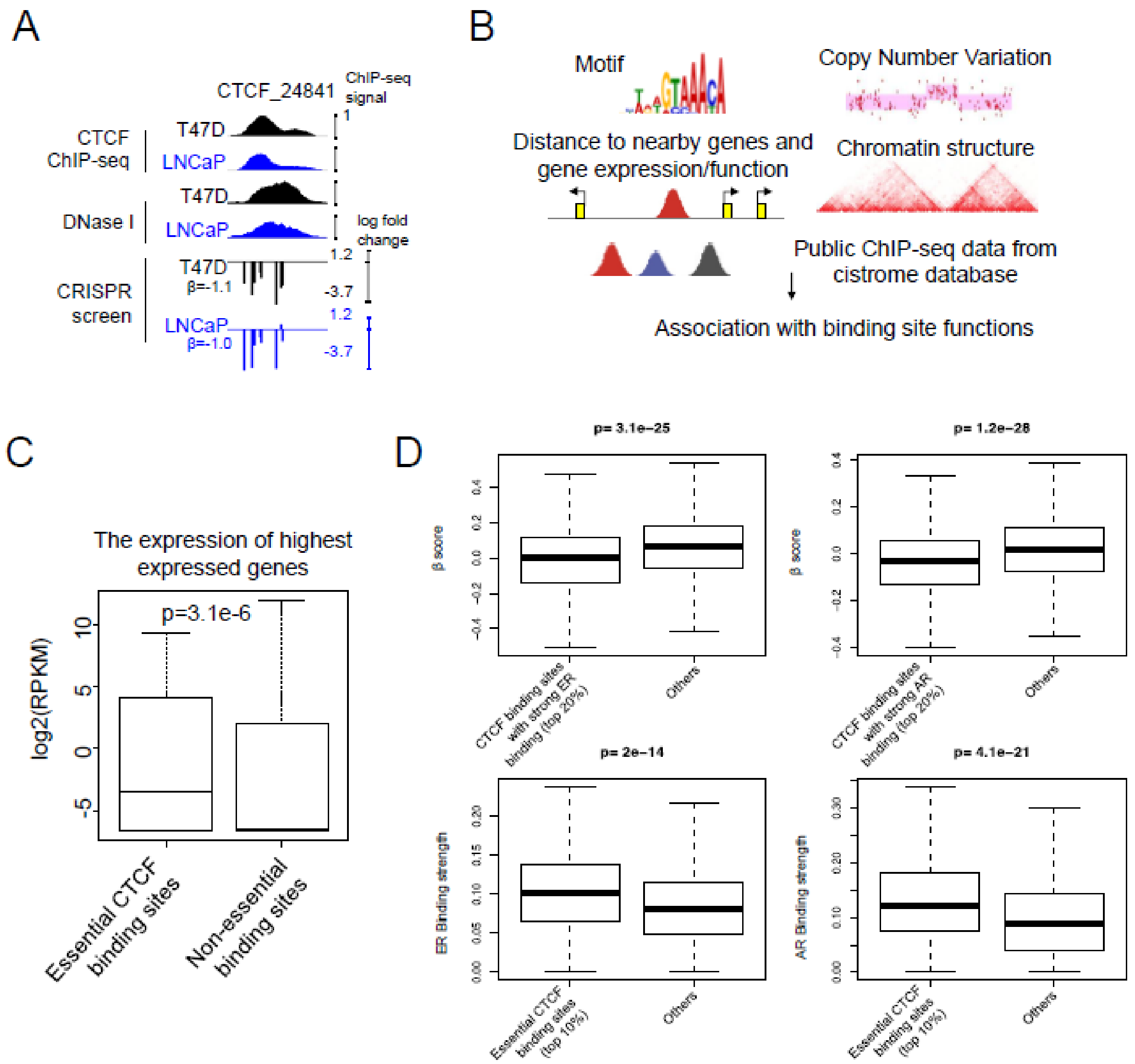

Figure S8. CTCF cistrome features.

(A) Chromatin structures of the top hit, CTCF_24841. (B) Possible features that are tested for the association with binding site functions in the screen. (C) The expressions of the highest expressed gene near essential CTCF binding sites and non-essential binding sites. Essential binding sites are defined as the 10% binding sites that have the smallest β scores in each screen. (D) The β score distribution of CTCF binding sites with strongest ER/AR bindings, and the ER/AR binding strengths of top essential CTCF binding sites vs. other binding sites in T47D or LNCaP cells.

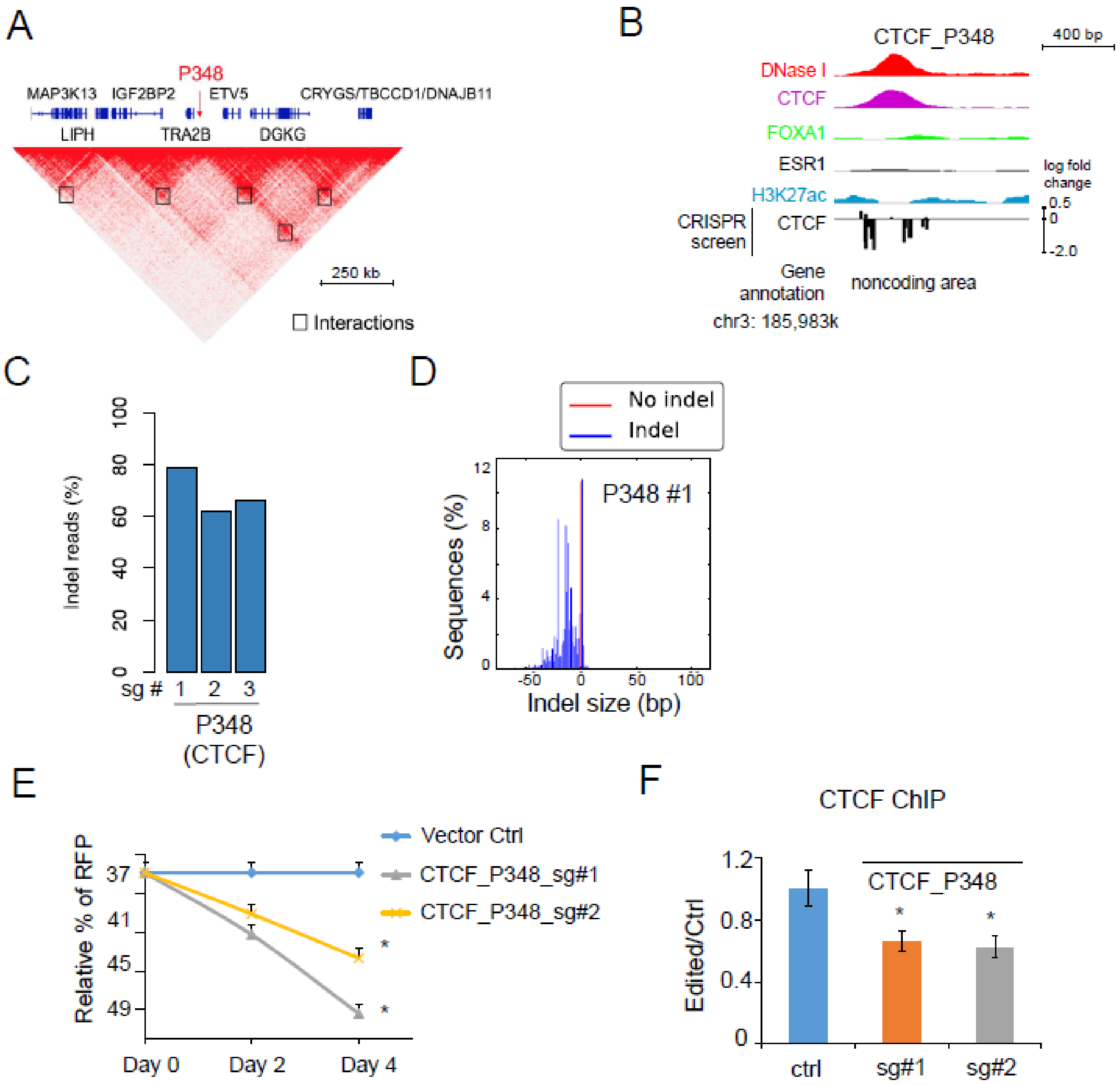

Figure S9. Experimental validation of top CTCF cistrome screening hits.
(A-B) One of the top essential CTCF binding site located at TAD boundary, CTCF_P348, is selected for further validation. The relative location of nearby genes, local TAD structures (A) and chromatin structures (B) are shown. (C) The percentage of indel reads (from targeted DNA sequencing) from CTCF_P348 loci using different sgRNAs. (D) The indel size distribution of knocking out CTCF_P348 locus using sgRNA #1. (E) Competitive cell growth assay confirmed the cell growth effect after knocking out CTCF_P348 using two individual sgRNAs. CRISPR-targeted T47D cells with indicated sgRNAs or control were mixed with red fluorescence protein (RFP)-expressing non-edited cells with a ratio around 2:1 at Day 0 and continually cultured for 4 days. RFP fraction was determined at indicated time points by flow cytometry. The more essential

of CRISPR-targeted site, the more percentage of RFP cells in the mixed population. *$P<0.05$. (F) The relative CTCF binding on CTCF_P348 after binding site knockout determined by ChIP-qPCR. *$P<0.05$.

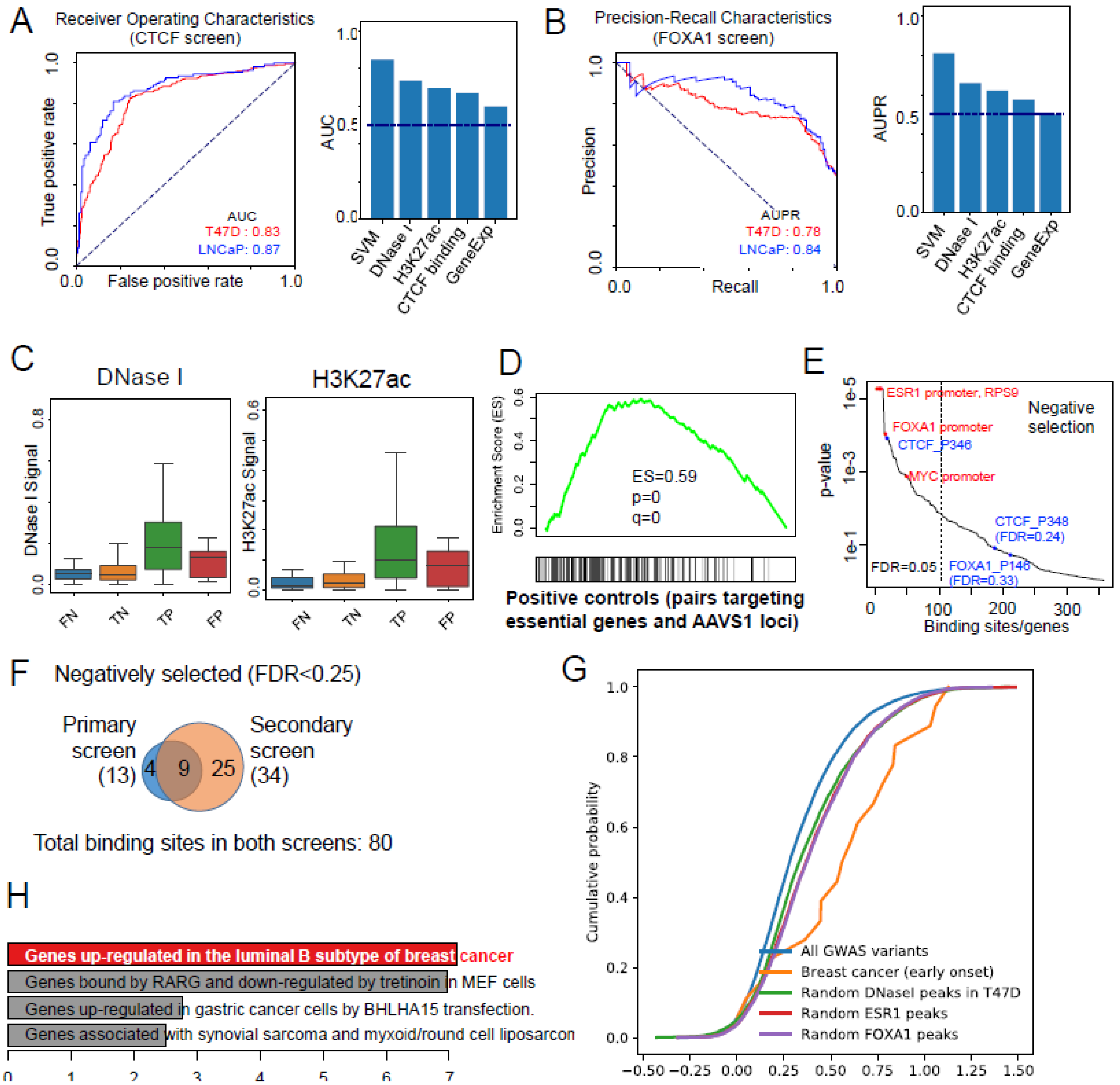

Figure S10. Prediction model and paired-gRNA screening analysis.
(A) The Receiver Operator Characteristic (ROC) curves of different approaches for predicting CTCF binding site essentialities using different combinations of features. The Area Under the Curve (AUC) values using SVM and individual features are also shown. (B) The Precision-Recall Characteristic (PR) curves of (A). The Area Under the PR curve (AUPR) of different approaches are shown. (C) The distribution of DNase I and H3K27ac signals in essential binding sites that are predicted as essential (TP or True Positive) or non-essential (FN or False Negative), as well as non-essential binding sites that are predicted as essential (FP or False Positive) or non-essential (TN or True Negative). (D) Gene Set Enrichment Analysis (GSEA) of positive control pairs (pairs targeting essential genes and AAVS1 loci) in ranked negative selection list of pgRNA screen. (E) Selected significant hits in the negative selection or positive selection of

pgRNA screen. Two binding sites selected for validation, as well as another CTCF binding site (CTCF_P346) that is 6 kb close to CTCF_P348 are also shown. (F) Overlap of binding sites with statistical significance in sgRNA and pgRNA screens. (G) The predicted essential score of different categories of enhancers. (H) The enriched terms (FDR<0.25) of genes that are close to enhancers associated with two traits, breast cancer (early onset) and breast cancer (survival). The GREAT prediction tool is used to identify genes near enhancers and enriched functional terms.

Supplementary Tables

Supplementary Table 1. MAGeCK results of genome-wide CRISPR gene screening on T47D and LNCaP cells.

Supplementary Table 2. The design of FOXA1 cistrome screening library.

Supplementary Table 3. The results of FOXA1 cistrome screening using MAGeCK-VISPR.

Supplementary Table 4. The design of CTCF cistrome screening library.

Supplementary Table 5. The results of CTCF cistrome screening using MAGeCK-VISPR.

Supplementary Table 6. Gene Set Enrichment Analysis (GSEA) results of CTCF_P348 knockout.

Supplementary Table 7. The design of paired-gRNA screening.

Supplementary Table 8. The results of paired-gRNA screening.

SUPPLEMENTARY METHODS

Cell Culture and Reagents

Breast cancer T47D cell were maintained in DMEM medium supplemented with 10% fetal bovine serum (FBS) as full media condition. When stimulated with estrogen 17β-estradiol (E2), T47D cells were cultured in phenol red-free DMEM medium with 10% charcoal/dextran-treated FBS for at least three days after switching from the full media. Prostate cancer LNCaP cells were cultured in RPMI 1640 media supplemented with 10% FBS as full media condition. HEK293FT cells were grown in DMEM medium with 10% FBS. The antibodies were purchased from the following companies: GAPDH (FL-335, Santa Cruz, Cat no. sc-25778), ERα (HC-20, Santa Cruz, Cat no. sc-543), FOXA1 (Abcam, Cat no. ab23738) and CTCF (EMD Millipore, Cat no. 07-729).

Cistrome-Targeting CRISPR Library Design

FOXA1 binding regions selection. We select two types FOXA1 binding sites in T47D cells for screening: binding sites near essential genes of T47D cells, and binding sites that have the strongest binding strengths in T47D cells. For binding sites near essential genes, we use the data from genome-wide CRISPR gene screens performed on T47D cells, and choose the top 1000 genes that have the smallest β scores as T47D cell essential genes. All 1122 FOXA1 binding sites that are within [-50 kb, +50 kb] of essential gene TSS are selected in T47D cells. For binding sites with strongest bindings, we choose 4988 binding sites that have the strongest FOXA1 bindings based on a public T47D FOXA1 ChIP-seq data**(3)**. Possible binding sites are further filtered out such that they do not overlap with any exons of coding genes, and do not fall within the 5 kb region upstream of the TSS of any coding genes.

CTCF binding region selection. Two different types of CTCF binding regions are selected, including "constitutive" regions and "unique" cell type-specific regions. Constitutive CTCF regions are regions that have CTCF bindings in at least 95% of the CTCF ChIP-seq samples in the cistrome database**(4)**. "Unique" regions that only have bindings in T47D or LNCaP cells are also selected. We first identified CTCF bindings in both cell lines from ENCODE consortium**(5)**. For T47D cell line, we select these regions such that no constitutive binding sites or binding sites in LNCaP cell line occur around the [-500 bp, +500 bp] region of the binding site in T47D cells (same criteria for selecting LNCaP cell unique regions). Possible binding sites are further filtered out such that they do not overlap with any exons of coding genes, and do not fall within the 5 kb region upstream of the TSS of any coding genes. We select the strongest 3740 constitutive binding sites, as well as 1008 and 816 unique binding sites for T47D and LNCaP cells for CRISPR targeting, respectively.

sgRNA selection. We design 20 sgRNAs targeting each binding site. For each binding site, we scan all possible 19-nt sgRNAs with canonical PAM motif that: (1) have the efficiency score >0, (2) are uniquely mapped to the genome with 1 mismatch tolerated, (3) have [5%-95%] GC content, (4) have <45% "G"s, and (5) are within the [-150 bp,

+150 bp] of the binding site summit. Only regions with at least 20 sgRNAs are selected. If there are more than 20 possible sgRNAs, the top 20 sgRNAs that are closest to the binding summit are selected. Overall, there are on average 19 sgRNAs/region for CTCF binding sites and 16 sgRNAs/region for FOXA1 binding sites. Most (99%) of the CTCF/FOXA1 binding sites are targeted by at least 10 sgRNAs.

Negative controls. Negative control sgRNAs are selected from whole genome human screening library. 267 AAVS1-targeting sgRNAs and 398 non-targeting sgRNAs are included.

Positive controls. Positive control sgRNAs are selected from whole genome human screening library. There are 730 positive control sgRNAs targeting essential genes (like ribosomal genes). Each essential gene is targeted by 5 sgRNAs.

CRISPR Library Synthesis and Construction

The pooled synthesized oligos were PCR amplified and then cloned into lentiCRISPRv2-puro vector via BsmBI site by Gibson Assembly. The ligated Gibson Assembly mix was transformed into self-prepared electrocompetent DH5α *E. coli* by electrotransformation to reach the efficiency with at least 20X coverage representation of each clone in the designed library. The transformed bacterial was cultured directly in liquid LB medium for 16~20 hours at low temperature 16℃ to minimize the recombination events in *E. coli*. The library plasmids were then extracted with GenElute^{TM} HP Endotoxin-Free Plasmid Maxiprep Kit (Sigma, Cat no. NA0410-1KT). To confirm the designed guide RNA sequences were successfully cloned into the plasmid library, we PCR amplified the guide RNA sequences, prepared sequencing libraries and employed Nextseq 500 sequencing platform to validate the inserted gRNA sequences as a stringent QC for the plasmid library. After alignment to our designed sequences, more than 99.92% of designed gRNA sequences were present in our plasmid libraries, indicating the high quality of the libraries.

Pooled Genome-wide CRISPR Screen

FOXA1 and CTCF cistrome-targeting plasmid libraries under lentiviral lentiCRISRPv2-puro backbone were firstly transfected along with pCMV8.74 and pMD2.G packaging plasmids into HEK293FT cells using X-tremeGENE™ HP DNA Transfection Reagent (Roche, Cat no. 6366236001) to generate CRISPR component-expressing lentivirus. Harvest virus-containing media at 48 hours and 72 hours post-transfection, and spin down the media at 1000 rpm for 5 min to remove the floating cells and cell debris. Carefully collect the virus supernatant, aliquot and store them at -80℃ for further use. Test the virus titer and MOI (multiplicity of infection) before proceeding to the genome-wide screen.

For full media screen, $1x10^8$ to $2x10^8$ T47D or LNCaP cells were infected with CTCF or FOXA1 cistrome-targeting lentiviral libraries with MOI ~0.3. Two days later, select the infected cells with puromycin (3.5μg/mL for T47D cells and 1.5μg/mL for LNCaP cells) for three days to get rid of any non-infected cells before changing back to normal media.

After two days recovery post puromycin selection, around half portion of cells (at least $3x10^7$ cells, ~300x coverage for each library) were collected as Day 0 sample and stored at -80℃ for later genomic DNA isolation. The rest half of cells were continually cultured until four weeks later before harvesting as the end point sample. For screens in T47D cells under vehicle and E2 condition, cells were cultured in either vehicle (ethanol) or 10 nM E2-containing white medium for additional five weeks after harvesting Day 0 sample. Genomic DNA from Day 0 and the end time point samples was extracted. The regions encompassing the gRNAs were firstly PCR-amplified for around 18-20 cycles with the following primer pair: lentiCRISPR_F1: 5'-AATGGACTATCATATGCTTACCGTAACTTGAAAGTATTTCG-3'; lentiCRISPR_RV2: 5'-TCTACTATTCTTTCCCCTGCACTGTACCTGTGGGCGATGTGCGCTCTG-3'. The second round of PCR were employed to attach the illumina adaptors and index for around 10-12 cycles with the following primers: Cri_libarary_F: 5'-AATGATACGGCGACCACCGAGATCTACACTCTTTCCCTACACGACGCTCTTCCGATCTTCTTGTGGAAAGGACGAAACACCG-3'; Index_R: 5'-CAAGCAGAAGACGGCATACGAGATGTGACTGGAGTTCAGACGTGTGCTCTTCCGATCTNNNNNNTCTACTATTCTTTCCCCTGCACTGTACC-3' (N(6) are the specific index sequences). These PCR products were gel purified and pooled for illumina sequencing at ~300X coverage depth (around 30 million reads) per sample on illumina Nextseq 500 sequencing platform with the following custom sequencing primers: Cri_lib_seq: 5'-GCTCTTCCGATCTTCTTGTGGAAAGGACGAAACACCG-3'; Cri_index_seq: 5'-CATCGCCCACAGGTACAGTGCAGGGGAAAGAATAGTAGA-3'. The data were analyzed by MeGeCK-VISPR.

Gene screens in T47D and LNCaP cells cultured under full medium were performed similarly as cistrome screens. The sgRNA library for gene screens targeting ~18,000 genes in the human genome was designed by our lab with an up-to-date algorithm to improve the specificity and efficacy of gRNAs and described in our recent studies**(6)**. Samples of Day 0 and Day 21 were used to quantify the gRNA abundance with similar library preparation protocol as cistrome screens. The data were analyzed by MeGeCK-VISPR**(7)**.

Genetic and epigenetic features associated with screening outcomes

We collected a set of genetic and epigenetic features in T47D and LNCaP. The H3K27ac and RNA-seq data was extracted from our previous studies**(8)**. Other ChIP-seq data was extracted from our cistrome database (cistrome.org), and only datasets that pass the quality control measurements in the database are used for downstream analysis. For each putative enhancer identified by DNase-I, the normalized ChIP-seq signals of the 150-bp window (centered on the DNase-I peak summit) were collected as features. For histone modification ChIP-seq data, the window size was extended to 300-bp.

Cloning of Individual CRISPR Vectors

Individual gRNA sequences were synthesized as short oligos and cloned into lentiCRISPRv2-puro vectors via BsmBI site. The gRNA target sequences are as follows: AAVS1: 5'- CTGGAAGATGCCATGACAGG-3'; CTCF_P348_sg1: 5'-ACACTGGGAACCGCCCAGG-3'; CTCF_P348_sg2: 5'-GATGAACCTGCAGTCCAGG-3'; CTCF_P348_sg3: 5'-AGTCCAGGAGGCCAAGGTC-3'; FOXA1_P146_sg1: 5'-GAAGCTTTCTAAGGCCTGG-3'; FOXA1_P146_sg2: 5'-CTGTTAAAGGAGCTATCCA-3'; FOXA1_sg1: 5'-TACTACGCAGACACGCAGG-3'; FOXA1_sg2: 5'-GACATGTTGAAGGACGCCG-3'; CTCF_sg1: 5'-CACAAGCGCACCCACACCG-3'; CTCF_sg2: 5'-AGCAAACTGCGTTATACAG-3'.

Deep Sequencing after CRISPR Mutagenesis

T47D cells were infected with the lentivirus packed with individual CRISPR-targeting vectors for CTCF_P348 (#1, #2 and #3) and FOXA1_P146 (#1 and #2). Two days post infection, puromycin (3. 5 µg/mL) was added to kill the non-infected cells for three days. The remaining cells were recovered in normal medium without puromycin for additional two days before splitting into 12-well plates. Cells were cultured for additional three days and then harvested for the genomic DNA (gDNA) extraction with the DNeasy Blood & Tissue Kit (Qiagen). The fragment surrounding the target region (100-200 bp amplicons) was firstly PCR-amplified for around 18-20 cycles with the following primer pair: CTCF_P348_F1: 5'-GCTCTTCCGATCTTCTTGTGGAAAGGACGAAACACCGAACAGCTATAATTATTGTTGAGC-3'; CTCF_P348_R1: 5'-TCTACTATTCTTTCCCCTGCACTGTACCCATCTAGTGGTGGGAGAAGGAAG-3'; CTCF_P348_F2: 5'-GCTCTTCCGATCTTCTTGTGGAAAGGACGAAACACCGGAGCATAATTCCTCCCCTTGCCT-3'; CTCF_P348_R2: 5'-TCTACTATTCTTTCCCCTGCACTGTACCCTTTCTTATGCTGGACTCATACT-3'; CTCF_P348_F3: 5'-GCTCTTCCGATCTTCTTGTGGAAAGGACGAAACACCGATTGTTGAGCATAATTCCTCCC-3'; CTCF_P348_R3: 5'-TCTACTATTCTTTCCCCTGCACTGTACCCTGGACTCATACTGCCATCTAG-3'; FOXA1_P146_F1: 5'-GCTCTTCCGATCTTCTTGTGGAAAGGACGAAACACCGCAGGAAGACATGGTATCAGGGTA-3'; FOXA1_P146_R1: 5'-TCTACTATTCTTTCCCCTGCACTGTACCAAATGGGCTTGAGTTCTTTAGC-3'; FOXA1_P146_F2: 5'-GCTCTTCCGATCTTCTTGTGGAAAGGACGAAACACCGATGCAGTGGGGACCTTAGGTCCT-3'; FOXA1_P146_R2: 5'-TCTACTATTCTTTCCCCTGCACTGTACCAACAGAGCTCTACAAAGCAGATG-3'. The second round of PCR were employed to attach the illumina adaptors and index for around 10-12 cycles with the following primers: Cri_libarary_F: 5'-AATGATACGGCGACCACCGAGATCTACACTCTTTCCCTACACGACGCTCTTCCGATCTTCTTGTGGAAAGGACGAAACACCG-3'; Index_R: 5'-CAAGCAGAAGACGGCATACGAGATGTGACTGGAGTTCAGACGTGTGCTCTTCCGATCTNNNNNNTCTACTATTCTTTCCCCTGCACTGTACC-3' (N(6) are the specific index

sequences). These PCR products were gel purified and pooled for illumina Miseq SE250 sequencing with the following custom sequencing primers: Cri_lib_seq: 5'-GCTCTTCCGATCTTCTTGTGGAAAGGACGAAACACCG-3'; Cri_index_seq: 5'-CATCGCCCACAGGTACAGTGCAGGGGAAAGAATAGTAGA-3'. The amplicon sequencing data was analyzed using CRISPResso**(9)**.

Competitive Cell Growth Assay

T47D cells infected with individual CRISPR-targeting virus for selected site (CTCF_P348 and FOXA1_P146) were firstly screened by puromycin (3. 5 μg/mL) for three days to remove non-infected cells and recovered in normal medium for additional two days before splitting. When splitting, these CRISPR-targeting cells were mixed with non-edited cells that stably expresses red fluorescence protein (RFP) at a ratio ~2:1 (RFP cells accounts for ~33% in the whole mixed populations). The mixed cells were continually cultured for four days. The RFP fraction was determined at Day 0, 2 and 4 by Flow Cytometry. The more essential of CRISPR-targeted site, the more percentage of RFP cells in the mixed population.

Chromatin Immunoprecipitation

T47D cells (control and indicated edited cells) grown in 15 cm dish in full media were crosslinked with 1% formaldehyde for 10 min at room temperature and then lysed with 500 μL lysis buffer (1% SDS, 10mM EDTA, 50mM Tris-HCl pH 8.1 plus protease inhibitors) for 10 min on ice. The cell lysate was then sonicated to break down genomic DNA into around 100-500 bp range before centrifugation at 14,000 rpm for 10 min at 4 ℃. Collect the supernatant containing the cell lysate and dilute with dilution buffer (1% triton, 2mM EDTA, 150mM NaCl, 20mM Tris-HCl pH 8.1) at 1: 10. Add protein G magnetic beads pre-bound to antibody of choice and incubate with rotation at 4℃ for at least 6 hours or overnight. Collect the beads and wash six times with RIPA buffer (50mM HEPES pH 7.6, 1mM EDTA, 0.7% Na Deoxycholate, 1% NP-40 and 0.5M LiCl) and two times with TE (pH 7.6). After removing the residual TE, add 100 μL of elution buffer (1% SDS and 0.1M $NaHCO_3$) and de-crosslink at 65℃ for 6 hours before purification with QIAquick PCR purification kit (Qiagen). The enrichment of CTCF or FOXA1 on the indicated sites was quantified by ChIP-qPCR using the following primers: CTCF_P348_ChIP_F: 5'-TGGACTGCAGGTTCATCTTG-3'; CTCF_P348_ChIP_R: 5'-TTGGCTTTATTCCCCAAAAA-3'; FOXA1_P146_F: 5'-GGTATAACTGAGAGCCTGATCCA-3'; FOXA1_P146_R: 5'-CAAAGCAGATGAAGCCAGCTA-3'.

RNA Extraction and qRT-PCR

RNA was extracted with RNeasy Mini Kit (Qiagen) following the manual instruction. Reverse transcription was performed with MultiScribe™ Reverse Transcriptase (ThermoFisher Scientific, Cat no. 4311235) to generate the random-primed first-strand complementary DNA (cDNA). Real time PCR was carried out on ABI Prism 7300 or 7500 systems with SYBR Green PCR master mix. The primers for qRT-PCR are as follows: RPS28_RT_F: 5'-CGATCCATCATCCGCAATG-3'; RPS28_RT_R: 5'-

AGCCAAGCTCAGCGCAAC-3'; ESR1_RT_F: 5'-GGGAAGTATGGCTATGGAATCTG-3'; ESR1_RT_R: 5'-TGGCTGGACACATATAGTCGTT-3'.

Small Interfering RNA (siRNA) Knockdown
T47D cells were seeded in 12-well plate under full media condition and transfected with 40 nM siRNA oligos by RNAiMax reagent (Life Technology, Cat no. 13778-150). Cells were harvested 72 hours post transfection to determine the knockdown efficiency and resulting effect on other proteins by Western blot analysis. The siRNA oligos were synthesized by Sigma and their target sequences are as follows: siControl: 5'-GCGACCAACGCCUUGAUUG-3'; siFOXA1_1#: 5'- CGUACUACCAAGGUGUGUA-3' and siFOXA1_2#: 5'- CACACAAACCAAACCGUCA-3'.

Paired Guide RNA Library Design
We designed a paired guide RNA library targeting two types of binding sites: selected binding sites in our primary CTCF or FOXA1 screens, and all DNase I binding sites that are <50 kb of known important genes in T47D cells. We select 66 binding sites with or without statistical significance in primary screens, as well as all DNase I binding sites near the following genes: ESR1, MYC, FOXA1, GATA3, known oncogenes in T47D cells; and PTEN, TSC1, RB1, CSK, known tumor suppressor genes in T47D cells. Negative control pairs where both gRNAs target the AAVS1 loci are included. Positive control pairs are included, where one gRNA targets known essential genes (as described in primary screening library design) and the other sgRNA targets the AAVS1 loci.

For the selected binding site, we first scan and filter all possible nearby sgRNAs, similar to the sgRNA design in primary screens. We then choose 5 sgRNAs on the left (or right) of the binding site summit, and are [75 bp, 150 bp] away from the summit. These 5X5 combinations lead to 25 pgRNAs with distance between 150-300bp, and cover the whole selected binding site.

Construction of pgRNA library
The construction of the pgRNA library was according to the methods as described previously(10).

Step I: pgRNA spacer cloning. The pooled oligonucleotide libraries were synthesized by Agilent Inc. Full-length oligonucleotides with paired-gRNA spacers (i.e., 19-nt sequences that target desired loci) were amplified by PCR with Q5 polymerase (NEB). PCR reactions were set up according to the standard manual protocol, with 2 μL of synthesized oligonucleotides as template (around 40 ng), an annealing temperature of 68 °C and an extension time of 20 s for 6 cycles. The primers were:

ARRAY_F, 5'-TAACTTGAAAGTATTTCGATTTCTTGGCTTTATATATCTTGTGGAAAGGACGAAACACCG-3'; ARRAY_R, 5'-

ACTTTTTCAAGTTGATAACGGACTAGCCTTATTTTAACTTGCTATTTCTAGCTCTAAA
AC-3’.

The 196-bp PCR product was purified by 2% agarose gel electrophoresis and the usage of a MinElute Gel Extraction Kit (Qiagen). Then, the lentiCRISPRv2-puro vector (Addgene #52961) was digested with BsmBI (NEB) according to the standard manual protocol and then purified with a DNA Clean & Concentrator-5 Kit (Zymo Research). To insert the paired-gRNAs into the vector, the Gibson assembly reactions were performed as follows: linearized lentiCRISPRv2 vector, 200 ng; dual-gRNA inserts, 40 ng; 2× Gibson Assembly Master Mix (NEB), 10 µl; H_2O up to 20 µL. After incubation at 50 °C for 1 h, the product was purified with a DNA Clean & Concentrator-5 Kit (Zymo Research) and then transformed into Endura electro-competent cells (Lucigen) with a Bio-Rad Electroporator. Four parallel transformations were performed to ensure adequate library representation. A small fraction (1-10 µL) of cultures was spread on ampicillin (100 µg/ml) containing LB plates to calculate the library coverage, and the rest of the cultures were amplified overnight in 150 ml LB medium; over 50X library coverage was ensured. The plasmid DNA was then extracted with a GenElute™ HP Endotoxin-Free Plasmid Maxiprep Kit (Sigma) and 10 independent clones were picked and Sanger-sequenced to estimate the overall quality of the library.

Step II: insertion of the first gRNA scaffold and the mouse U6 *promoter.* The step 1 library plasmids were digested with BsmBI (NEB) followed by the treatment with 2 µL of calf intestinal alkaline phosphatase (NEB) at 37 °C for 30 min, and cut plasmids were gel-purified through agarose gel electrophoresis and MinElute Gel Extraction Kit (Qiagen). At the same time, the amplified step 2 insert was digested by BsmBI and purified by Gel Purification. The sequence of the step 2 insert, with the left gRNA scaffold underlined and m*U6* promoters in bold, was

5’-
ACTGACGTCTCAGTTTAAGAGCTAAGCTGGAAACAGCATAGCAAGTTTAAATAAGG
CTAGTCCGTTATCAACTTGAAAAAGTGGCACCGAGTCGGTGCTTTTTTTCTCGAGT
ACTAGGATCCATTAGGCGGCCGCGTCGACAAGCTTTCTAGAGAATTCGATCCGAC
GCGCCAT**CTCTAGGCCCGCGCCGGCCCCCTCGCACGGACTTGTGGGAGAAGCTC
GGCTACTCCCCTGCCCCGGTTAATTTGCATATAATATTTCCTAGTAACTATAGAGG
CTTAATGTGCGATAAAAGACAGATAATCTGTTCTTTTTAATACTAGCTACATTTTA
CATGATAGGCTTGGATTTCTATAACTTCGTATAGCATACATTATACGAAGTTATAA
ACAGCACAAAAGGAAACTCACCCTAACTGTAAAGTAATTGTGTGTTTTGAGACTA
TAAGTATCCCTTGGAGAACCACCTTGTTG**GGAGACGACTGA-3’

Subsequently, the following ligation reaction was set up, followed by overnight incubation at 16 °C and subsequent heat inactivation at 65 °C for 10 min: 10× T4 DNA ligase buffer, 2 µL; step 1 library, digested, 100 ng; step 2 insert, digested, 100 ng; T4 DNA ligase (high concentration), 1 µL; H_2O up to 20 µL. 2 uL of the reaction product was transformed into Endura electro-competent cells (Lucigen) according to the manufacturer’s protocol, with a Bio-Rad Electroporator. A small fraction (1–10 µL) of

cultures was spread on ampicillin (100 μg/ml) containing LB plates to calculate the library coverage, and the remainder was plated on 245 mm^2 LB-ampicillin plates and grown overnight at 37 °C for amplification. Four transformations were required to obtain 50X library coverage. The plasmid DNA was extracted with a GenElute™ HP Endotoxin-Free Plasmid Maxiprep Kit (Sigma). Library diversity was determined by next generation sequencing.

NGS library preparation and sequencing of pgRNA library.

Harvested cell pellets were stored at −80 °C until extraction of genomic DNA with a DNeasy Blood and Tissue Kit (Qiagen). The pgRNA cassette was amplified and prepared for deep sequencing through two rounds of PCR. The first round PCR was performed as ten separate 50-μL reactions with 2 μg input genomic DNA or 50 ng plasmid DNA per reaction. The PCR primers were as follows:
pgRNA_Lib_F, 5’-AATGATACGGCGACCACCGAGATCTACACTCTTTCCCTACACGACGCTCTTCCGATCTTTGTGGAAAGGACGAAACACCG-3’;
pgRNA_Lib_R1, 5’-TCTACTATTCTTTCCCCTGCACTGTACCCGGACTAGCCTTATTTTAACTTGCTATTTCTAGCTCTAAAAC-3’.

The numbers of cycles were tested to ensure the effective amplification. Amplicons (700 bp) of multiple reactions for each sample were pooled, size-selected and purified with Agencourt AMPure XP beads at a ratio ~0.8. The second round PCR was performed with separate 50 μL reactions with 5 ng of first-step PCR product per reactions using primers:

pgRNA_Lib_F, 5’-AATGATACGGCGACCACCGAGATCTACACTCTTTCCCTACACGACGCTCTTCCGATCTTTGTGGAAAGGACGAAACACCG-3’;
pgRNA_Lib_R2, 5’-CAAGCAGAAGACGGCATACGAGATGTGACTGGAGTTCAGACGTGTGCTCTTCCGATCTNNNNNNNNTCTACTATTCTTTCCCCTGCACTGTACC-3’ ($N_{(8)}$ is the specific index sequences), and purified by gel purification kit. The amplified pgRNA library was then be sequenced using the illumina MiSeq sequencing platform with the customized sequencing primers as follows:

read1_seq: GAGATCTACACTCTTTCCCTACACGACGCTCTTCCGATCT (for the 1st gRNA)
read2_seq: TGCACTGTACCCGGACTAGCCTTATTTTAACTTGCTATTTCTAGCTCTAAAAC (for the 2nd gRNA)
index_seq: GCTAGTCCGGGTACAGTGCAGGGGAAAGAATAGTAGA.

Predicting Enhancer Functions

For building machine learning models to predict enhancer functions, we use both essential and non-essential sites in the screening as training samples. Since the number of significant sites is few, we increase the threshold (negative rank < 300) to select more (but less statistically significant) sites as essential sites. Non-essential sites are chosen such that they are neither negatively nor positively selected (p>0.5), and their absolute log fold change is less than 0.1. In all the datasets, essential and non-essential sites are balanced (essential to non-essential rate is between 0.85 and 1.1).

The SVM toolkit in the scikit-learn package (https://scikit-learn.org) was used for training and prediction. We used a genetic algorithm combined with SVM (GA-SVM) to select best feature combinations(11, 12). Briefly, GA-SVM is an iterative process, where a set of feature combinations are subjected to randomly adding/removing/changing one feature at each iteration. Features that reach better prediction performance have higher chance to go to the next iteration. This process is repeated several times to select the best combination of features. The entire dataset was split into training and validation set, where training dataset was used to train SVM, and the AUROC value calculated on validation set was used to evaluate feature combinations.

GWAS associated SNPs and their traits are downloaded from GWAS Catalog website (https://www.ebi.ac.uk/gwas/). If the location of the SNP overlaps with known DNase I peak in T47D or LNCaP, the corresponding DNase I binding site will serve as the SNP-bearing enhancer. If no DNase I peak overlaps with the SNP location, we will search for possible FOXA1, ER or GATA3 binding sites. If none of these peaks overlap with SNP, we will consider a 150-bp window centered on that SNP as an "enhancer" for downstream analysis. The prediction algorithm was applied to evaluate whether these SNP-associated enhancers are essential.

1. Aguirre AJ, et al. (2016) Genomic copy number dictates a gene-independent cell response to CRISPR-Cas9 targeting. *Cancer Discov*:CD–16–0154.

2. Hart T, Brown KR, Sircoulomb F, Rottapel R, Moffat J (2014) Measuring error rates in genomic perturbation screens: gold standards for human functional genomics. *Mol Syst Biol* 10(7):733–733.

3. Hurtado A, Holmes KA, Ross-Innes CS, Schmidt D, Carroll JS (2011) FOXA1 is a key determinant of estrogen receptor function and endocrine response. *Nat Genet* 43(1):27–33.

4. Liu T, et al. (2011) Cistrome: an integrative platform for transcriptional regulation studies. 12(8):R83.

5. ENCODE Project Consortium (2012) An integrated encyclopedia of DNA elements in the human genome. *Nature* 489(7414):57–74.

6. Chen C-H, et al. (2018) Improved design and analysis of CRISPR knockout screens. *Bioinformatics* 6:914.

7. Li W, et al. (2015) Quality control, modeling, and visualization of CRISPR screens with MAGeCK-VISPR. 16(1):281.

8. Xiao T, et al. (2018) Estrogen-regulated feedback loop limits the efficacy of estrogen receptor-targeted breast cancer therapy. *Proceedings of the National Academy of Sciences of the United States of America* 115(31):7869–7878.

9. Pinello L, et al. (2016) Analyzing CRISPR genome-editing experiments with CRISPResso. *Nat Biotechnol* 34(7):695–697.

10. Zhu S, et al. (2016) Genome-scale deletion screening of human long non-coding RNAs using a paired-guide RNA CRISPR-Cas9 library. *Nat Biotechnol*. doi:10.1038/nbt.3715.

11. Liu JJ, et al. (2005) Multiclass cancer classification and biomarker discovery using GA-based algorithms. *Bioinformatics* 21(11):2691–2697.

12. Alba E, Garcia-Nieto J, Jourdan L, Talbi E-G (2007) Gene selection in cancer classification using PSO/SVM and GA/SVM hybrid algorithms (IEEE), pp 284–290.
